# SMYD3-mediated lysine methylation in the PH domain is critical for activation of AKT1

**DOI:** 10.18632/oncotarget.11898

**Published:** 2016-09-08

**Authors:** Yuichiro Yoshioka, Takehiro Suzuki, Yo Matsuo, Makoto Nakakido, Giichiro Tsurita, Cristiano Simone, Toshiaki Watanabe, Naoshi Dohmae, Yusuke Nakamura, Ryuji Hamamoto

**Affiliations:** ^1^ Section of Hematology/Oncology, Department of Medicine, The University of Chicago, MC2115 Chicago, IL 60637, USA; ^2^ Biomolecular Characterization Unit, RIKEN Center for Sustainable Resource Science, Wako, Saitama 351-0198, Japan; ^3^ OncoTherapy Science, Inc., Takatsu-ku, Kawasaki, Kanagawa 213-0012, Japan; ^4^ Department of Surgery, IMSUT Hospital, Institute of Medical Science, The University of Tokyo, Minato-ku, Tokyo 108-8639, Japan; ^5^ Division of Medical Genetics, Department of Biomedical Science and Human Oncology (DIMO), University of Bari ‘Aldo Moro’, Bari 70124, Italy; ^6^ Department of Surgical Oncology, Graduate School of Medicine, The University of Tokyo, Bunkyo-ku, Tokyo 113-8654, Japan

**Keywords:** SMYD3, AKT1, lysine methylation, PH domain, human cancer

## Abstract

AKT1 is a cytosolic serine/threonine kinase that is overexpressed in various types of cancer and has a central role in human tumorigenesis. Although it is known that AKT1 is post-translationally modified in various ways including phosphorylation and ubiquitination, methylation has not been reported so far. Here we demonstrate that the protein lysine methyltransferase SMYD3 methylates lysine 14 in the PH domain of AKT1 both *in vitro* and *in vivo*. Lysine 14-substituted AKT1 shows significantly lower levels of phosphorylation at threonine 308 than wild-type AKT1, and knockdown of SMYD3 as well as treatment with a SMYD3 inhibitor significantly attenuates this phosphorylation in cancer cells. Furthermore, substitution of lysine 14 diminishes the plasma membrane accumulation of AKT1, and cancer cells overexpressing lysine 14-substiuted AKT1 shows lower growth rate than those overexpressing wild-type AKT1. These results imply that SMYD3-mediated methylation of AKT1 at lysine 14 is essential for AKT1 activation and that SMYD3-mediated AKT1 methylation appears to be a good target for development of anti-cancer therapy.

## INTRODUCTION

v-Akt Murine Thymoma Viral Oncogene Homolog 1 (AKT1), a serine/threonine-protein kinase also known as protein kinase B Alpha (PKB Alpha), is a key mediator of a signaling pathway that governs various cellular processes regulating cell growth, survival, glucose metabolism, genome stability and neovascularization [1]. The activation of AKT1 is induced by its membrane localization, which is initiated by the binding of the pleckstrin homology (PH) domain to phosphatidylinositol-3,4,5-triphosphate (PtdIns(3,4,5)P3) or phosphatidylinositol-3,4-bisphosphate (PtdIns(3,4)P2), and triggers phosphorylation of the regulatory amino acids, threonine 308 (Thr 308) and serine 473 (Ser 473) [2, 3]. It is known that dysregulation of AKT1 activity is one of the critical factors for the development and/or progression of a variety of human cancers [4, 5]. The oncogenic activation of AKT1 is driven by several means, most commonly either by the compromise in its membrane-targeting by PH domain or by the pathological structural changes caused by somatic mutation [2], which were identified in a subset of human carcinomas [6, 7]. This mutation of glutamic acid with lysine (E17K) at codon 17 alters the lipid-binding specificity of AKT1, which leads to enhancement of its membrane association and constitutive activation of AKT1 signaling [6–8]. Additionally, two promising anti-cancer agents targeting AKT1 (the cytotoxic alkylphospholipids Perifosine and Miltefosine) bind to the PH domain, which blocks membrane localization of AKT1 and results in a reduction in the level of AKT1 phosphorylation [9]. These results reveal that the PH domain on AKT1 plays a critical role in the regulation of its activity.

SET and MYND domain-containing protein 3 (SMYD3) is a protein lysine methyltransferase, involved in colorectal cancer, hepatocellular carcinoma and breast cancer [10–14]. In addition to histone proteins, SMYD3 methylates Vascular Endothelial Growth Factor Receptor 1 (VEGFR1) and Mitogen Activated Protein Kinase 2 (MAP3K2) [15, 16]. Accumulated evidence suggests SMYD3 is now considered to play a fundamental role in human tumorigenesis [17–21]. In the present study, we demonstrate that SMYD3 methylates lysine residues in the PH domain of AKT1, and this methylation is essential for AKT1 activation in human cancer cells. Our results explored a novel mechanism relevant to activation of the AKT1 signaling pathway, and it may be an appropriate strategy to target SMYD3-mediated AKT1 methylation for the development of anti-cancer treatment.

## RESULTS

### SMYD3 methylates lysine residues in the PH domain of AKT1 *in vitro*

**Figure 1:**
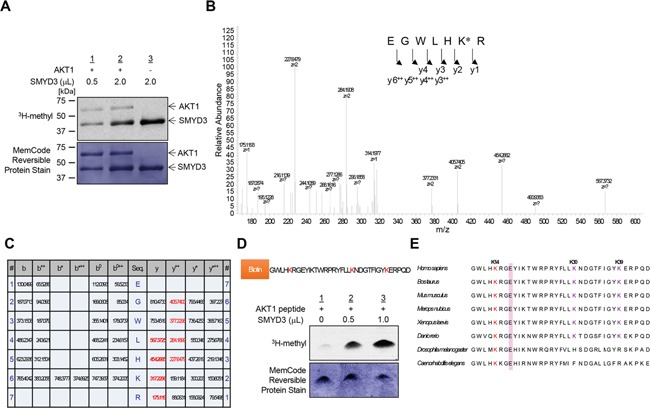
SMYD3 methylates AKT1 *in vitro* **A.** Recombinant AKT1 protein was incubated with different concentration of SMYD3 in the presence of S-adenosyl-L-[methyl-^3^H]-methionine, and methylation signal was detected by autoradiography (upper panel). Amounts of loading proteins were evaluated by staining with MemCode™ Reversible Protein Stain (lower panel). The concentration of SMYD3 is 11.5 μM. **B.** The LC-MS/MS spectrum corresponding to the monomethylated AKT1 9-15 peptide. AKT1 recombinant protein was incubated with SMYD3 and S-adenosyl-L-methionine, followed by separation by SDS-PAGE. An excised AKT1 band from the gel was digested with trypsin and subjected to LC-MS/MS analysis. The methylation site was determined by MASCOT search. The 14 Da increase of the Lys 14 residue was observed. **C.** The theoretical values of MS/MS fragments ions of the Lys 14 monomethylated AKT1 9-15 peptides are summarized in the table. The abbreviations of fragment ion types were indicated by the MASCOT program (http://www.matrixscience.com/help/fragmentation_help.html). The observed ions in Figure 1B were indicated in red letters. **D.** The biotin-conjugated AKT1 10-44 peptide was incubated with SMYD3 in the presence of S-adenosyl-L-[methyl-^3^H]-methionine, and methylation signal was detected by autoradiography. Amounts of loading proteins were evaluated by staining with MemCode™ Reversible Protein Stain. **E.** Amino acid sequence alignment of AKT1. Lys 14 is highlighted in red and conserved among various species. Glu 17, which forms an ionic interaction with Lys 14, is shown covering a red hatched rectangle.

To investigate a possibility that AKT1 serves as a substrate for any protein methyltransferase(s) activity, we conducted an *in vitro* methyltransferase assay using the protein lysine methyltransferase SMYD3, Protein Arginine Methyltransferase 1 (PRMT1) and Protein Arginine Methyltransferase 5 (PRMT5), which are indicated as oncogenic protein methyltransferases and localized in the cytoplasm, as enzyme sources. We found that SMYD3 methylated AKT1 in a dose-dependent manner (Supplementary Figure S1 and Figure 1A). We previously reported that SMYD3 is overexpressed in colorectal, hepatocellular and breast cancers, and plays a critical role in tumorigenesis [10, 12]. According to the Oncomine database, SMYD3 is also overexpressed in esophageal squamous cell carcinoma, oral cavity squamous cell carcinoma, acute myeloid leukemia, pancreatic ductal adenocarcinoma, leiomyosarcoma and renal Wilms' tumor (Supplementary Figure S2). In addition, the Cancer Genome Atlas (TCGA) database indicates frequent amplification of SMYD3 in various types of cancer (Supplementary Figure S3). To further explore the biological functions of SMYD3 in human cancer, we performed liquid chromatography tandem-mass spectrometry (LC-MS/MS) analysis of AKT1 protein and identified that lysines 14, 30 and 39 (Lys 14, Lys 30 and Lys 39) in the PH domain of AKT1 were possibly methylated by SMYD3 (Figure 1B-1C and Supplementary Figures S4, S5 and S6). To verify the methylation in this region, we prepared the peptide that included methylation sites, and conducted an *in vitro* methyltransferase assay. As shown in Figure 1D, the AKT1 peptide including three candidate lysines was methylated by SMYD3. Alignment of the PH domain of AKT1 protein showed that these methylation sites are conserved among various species, and in particular, Lys 14 is well conserved from *Caenorhabditis elegans* to *Homo sapiens* (Figure 1E), implying the importance of Lys 14 for AKT1 functions. Furthermore, glutamic acid 17 (Glu 17), which occupies the phosphoinositide-binding pocket and has a pivotal role in AKT1 activation [22, 23], is also well conserved among species, and basic Lys 14 is considered to form an ionic interaction with acidic Glu 17 in this pocket.

### SMYD3-mediated Lys 14 methylation activates phosphorylation of Thr 308 on AKT1

**Figure 2:**
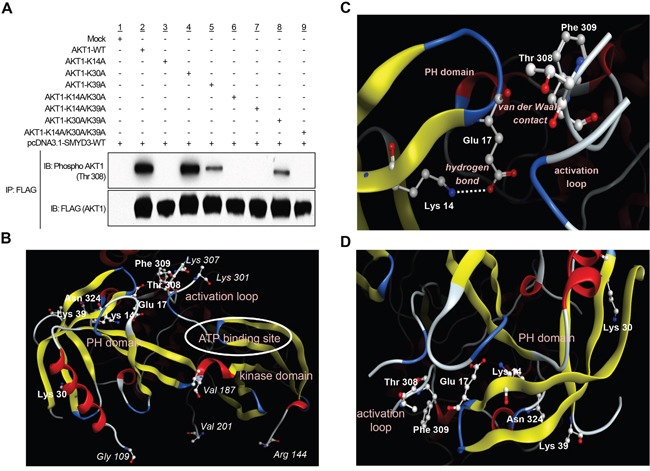
SMYD3-mediated lysine 14 methylation is critical for AKT1 activation **A.** 293T cells were transfected with FLAG-tagged wild-type AKT1 (AKT1-WT) or mutant-type AKT1 (AKT1-K14A, AKT1-K30A, AKT1-K39A, AKT1-K14A/K30A, AKT1-K14A/K39A, AKT1-K30A/K39A or AKT1-K14A/K30A/K39A) in the presence of a wild-type SMYD3 expression vector (pcDNA-SMYD3.1-WT). After 48 hours of incubation, cells were treated with 100 ng/ml of EGF, and then lysed with CelLytic™ M mammalian cell lysis/extraction reagent containing a protease inhibitor cocktail and a phosphatase cocktail, followed by immunoprecipitation using anti-FLAG^®^ M2 affinity gel. Immunoprecipitates were immunoblotted with anti-phospho AKT (Thr 308) and anti-FLAG (internal control) antibodies. **B.** The three-dimensional coordinate data for AKT1 were derived from the Protein Data Bank (entry code, 4EJN) [59]. PH domain, activation loop, kinase domain and ATP binding site are described. **C.** Lysine 14 methylation can conformationally affect threonine 308. Lys 14 is hydrogen-bounding to Glu 17. The distance between the side-chain amino nitrogen atom (Nζ) of Lys 14 and the side-chain carboxyl oxygen atom (Oε2) of Glu 17 is 3.17 Å. Glu 17 is in van der Waals contact with the activation loop (Thr 291 – Glu 314), on which Thr 308 is located. The distance between their closest atoms (the backbone carboxyl oxygen atom of Glu 17 and the side-chain β carbon atom of Phe 309) is 3.35 Å. A part of the activation loop (Asp 302 – Met 306) is missing in the crystal structure. The drawing was created using *Molecular Operating Environment* (MOE), 2014.09 (Chemical Computing Group Inc., Canada). **D.** The distance between Lys 14, Lys 30 or Lys 39 in the PH domain and activation loop is described.

Given that the PH domain on AKT1 protein plays a fundamental role in AKT1 activity through interaction with PtdIns(3,4,5)P3 or PtdIns(3,4)P2 [9], we attempted to elucidate whether SMYD3-mediated methylation affects AKT1 activity in cancer cells. To examine the direct effects of SMYD3-mediated methylation on AKT1 activity, we prepared seven AKT1 expression vectors that possess substitution of one or multiple methylation candidate sites (Lys 14, Lys 30 and Lys 39). We found that among the seven clones examined, four plasmids clones, commonly containing the K14A substitution, showed almost complete loss of AKT1 phosphorylation at Thr 308, which is the major indicator of AKT1 activity [24, 25], implying that Lys 14 methylation by SMYD3 appears to be essential for Thr 308 phosphorylation of AKT1 (Figure 2A). In addition to Lys 14, Lys 39-substituted AKT1 showed modest attenuation of Thr 308 phosphorylation while no effect was observed in the clone in which Lys 30 was substituted. The three dimensional structural analysis of AKT1 indicates that the distances between Thr 308 and each of these three methylation sites (Cα interatomic distance) are as follows: Lys 14-Thr 308, 15.8 Å, Lys 30-Thr 308, 30.5 Å and Lys 39-Thr 308, 22.7 Å (Figure 2B). An X-ray crystal structure analysis of AKT1 in Figure 2C further supports that Lys 14 and Thr 308 can indirectly associate with each other intramolecularly in the AKT1 protein [6, 22]. The side-chain amino group of Lys 14 is hydrogen-bonding to the side-chain carboxyl group of Glu 17, which is in van der Waals contact with the activation loop, on which Thr 308 is located. The methylation of Lys 14 substitutes a hydrophobic methyl group for a polar hydrogen molecule in the side-chain amino group of Lys 14. This should inevitably weaken the electrostatic interaction between Lys 14 and Glu 17. The resultant reduction of the positional constraint on Glu 17 imposed by Lys 14 would then increase the conformational flexibility of the Glu 17-containing turn structure (Figure 2C). It is therefore possible that the methylation of Lys 14, via the change in the interaction with Glu 17, is likely to cause a conformational change in the activation loop and then affect the phosphorylation status of Thr 308. Since the distance between Cβ atom in Lys 39 and Oδ atom in asparagine 324 (Asn 324) is 3.89 Å, these molecules are likely to be in van der Waals contact, and there is the electrostatic attraction between side chains of them (Figure 2D and Supplementary Figure S7). Hence, the methylation of Lys 39 may also affect the interaction between Lys 39 and Asn 324. However, because K14-substituted AKT1 showed complete loss of phosphorylation at Thr 308, we focused on Lys 14 methylation for further functional analyses.

### *In vivo* methylation of Lys 14 on AKT1 by SMYD3

**Figure 3:**
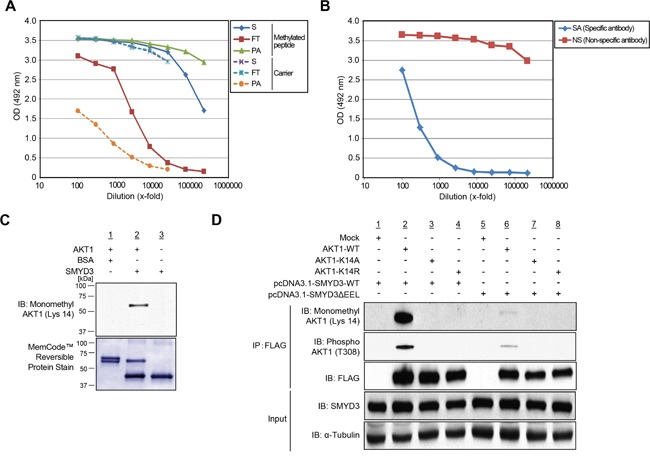
Validation of methylation on AKT1 at lysine 14 by specific antibody **A.** The enzyme-linked immunosorbent assay of the anti-monomethyl AKT1 (Lys 14) antibody. A rabbit was immunized with synthetic peptides, including Lys 14 monomethylation, and the affinity purification was carried out against the methylated synthetic peptides. The original serum (S) and the flow through (FT) show equal reactivity against the carrier protein. The purified antibody (PA) shows a stronger reactivity against the methylated peptide than the serum (S). **B.** Testing of specific antibody (SA) and non-specific antibody (NS) against the unmodified peptide by enzyme-linked immunosorbent assay. **C.** Validation of the anti-K14 monomethylated AKT1 antibody. Recombinant AKT1 protein or BSA and S-adenosyl-L-methionine were incubated in the presence or absence of recombinant SMYD3, and the reaction products were analyzed by SDS-PAGE, followed by western blot analysis using the anti-K14 monomethylated AKT1 antibody. The nitrocellulose membrane was stained with MemCode™ Reversible Stain Kit after western blot analysis. **D.** 293T cells were co-transfected with Mock, FLAG-AKT1-WT, FLAG-AKT1-K14A or FLAG-AKT1-K14R together with a wild-type SMYD3 expression vector (pcDNA3.1-SMYD3-WT) or an enzyme-inactive SMYD3 expression (pcDNA3.1-SMYD3-ΔEEL). After 48 hours of incubation, cells were treated with 100 ng/ml of EGF for 5 min, and then lysed with CelLytic™ M mammalian cell lysis/extraction reagent containing a protease inhibitor cocktail and a phosphatase cocktail. The samples were immunoblotted with anti-K14 monomethylated AKT1, anti-phospho AKT (Thr 308) and anti-FLAG antibodies after immunoprecipitating with anti-FLAG^®^ M2 affinity gel.

To confirm SMYD3-mediated Lys 14 methylation on AKT1 *in vivo*, we transfected wild-type FLAG-AKT1, with the SMYD3 expression vector, and conducted LC-MS/MS analysis of a protein immunoprecipitated with anti-FLAG^®^ M2 affinity gel (Supplementary Figure S8). Consequently, we confirmed monomethylation of AKT1 at Lys 14 *in vivo* (Supplementary Figure S8). To further validate *in vivo* methylation of AKT1 at Lys 14, we generated a specific antibody that recognizes Lys 14-monomethylated AKT1. Enzyme-linked immunosorbent assays (ELISAs) (Figure 3A, 3B) as well as an *in vitro* methyltransferase assay and subsequent western blot analysis (Figure 3C) revealed high specificity of anti-K14 monomethylated AKT1 antibody. To further investigate *in vivo* methylation of AKT1, we expressed FLAG-tagged wild-type AKT1 (AKT1-WT), or K14A- or K14R-substituted AKT1 proteins with a wild-type SMYD3 expression vector (SMYD3-WT) or an enzyme-inactive SMYD3 mutant (SMYD3ΔEEL) expression vector, followed by immunoprecipitation using anti-FLAG^®^ M2 affinity gel. Subsequent western blot analysis showed that the both Lys 14 monomethylation and Thr 308 phosphorylation signals of AKT1 were significantly attenuated in both K14A- and K14R-substituted AKT1 (Figure 3D), and that the enzyme-inactive SMYD3 mutant remarkably diminished Thr 308 phosphorylation signals of AKT1 (Figure 3D). These results suggest that SMYD3-mediated Lys 14 methylation of AKT1 is clearly observed *in vivo* and pivotal for phosphorylation of Thr 308 on AKT1.

### SMYD3-mediated Lys 14 methylation activates the AKT pathway in cancer cells

**Figure 4:**
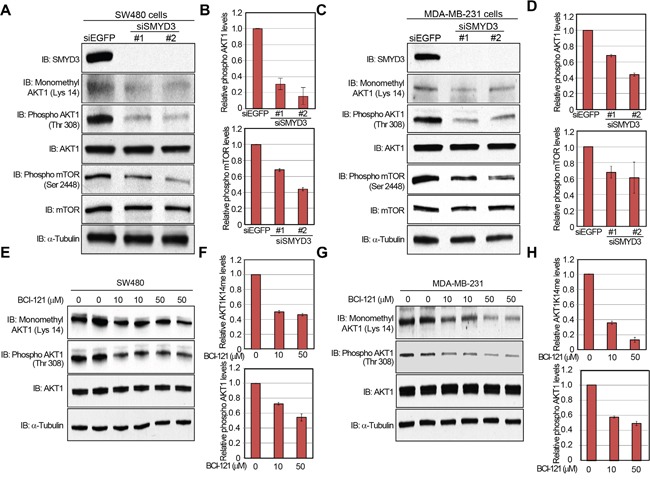
Knockdown and enzyme inhibitory of SMYD3 attenuate AKT1 activity **A, C.** Effects of SMYD3 knockdown on the AKT pathway in SW480 cells (A) and MDA-MB-231 cells (C). Cells were transfected with one control siRNA (siEGFP) or two SMYD3 siRNAs (#1 and #2), and after 72 hours of incubation, cells were treated with 100 ng/ml of EGF (SW480) or 100 ng/ml of NRG1 (MDA-MB-231). Then cells were lysed with CelLytic™ M mammalian cell lysis/extraction reagent containing a protease inhibitor cocktail and a phosphatase cocktail, followed by SDS-PAGE. Western blot analysis was performed using anti-SMYD3, anti-K14 monomethylated AKT1, anti-phospho AKT1 (Thr 308), anti-AKT1, anti-phospho mTOR (Ser 2448), anti-mTOR and anti-α-Tubulin (internal control) antibodies. **B, D.** X-ray films were scanned with GS-800™ calibrated densitometer (Bio-Rad). The intensity of phosphorylated AKT (Thr 308) and phosphorylated mTOR (Ser 2448) levels in SW480 cells (B) and MDA-MB-231 cells (D) was normalized by each total protein level. **E, G.** Effects of BCI-121 treatment on the AKT1 activity. SW480 cells (E) and MDA-MB-231 cells (G) were treated with 0, 10 or 50 μM of BCI-121. After 72 hours of incubation, cells were treated with 100 ng/ml of EGF (SW480) or 100 ng/ml of NRG1 (MDA-MB-231), and then lysed with CelLytic™ M mammalian cell lysis/extraction reagent containing a protease inhibitor cocktail and a phosphatase cocktail. Cell extracts were immunoblotted with anti-K14 monomethylated AKT1, anti-phospho AKT (Thr 308), anti-AKT and anti-α-Tubulin (internal control) antibodies. **F, H.** X-ray films were scanned with GS-800™ calibrated densitometer (Bio-Rad). The intensity of methylated AKT1 (Lys 14) and phosphorylated AKT1 (Thr 308) levels in SW480 cells (F) and MDA-MB-231 cells (H) was normalized by each total protein level.

A large body of literature and databases have documented frequent overexpression of SMYD3 (Supplementary Figures S2 and S3) and hyperactivation of the AKT pathway in a variety of malignancies [1, 11, 26]. Therefore, we examined the biological significance of SMYD3 on the AKT pathway in cancer cells. We knocked down SMYD3 in cancer cells by specific siRNAs and examined phosphorylation status of AKT1. As shown in Figure 4A and 4B, the phosphorylation level of AKT1 at Thr 308 was significantly diminished after knockdown of SMYD3 in the human colon cancer SW480 cells. Consistently, phosphorylation levels of mTOR, which is a major physiological substrate of AKT1, were also decreased (Figure 4A, 4B). The similar results were obtained when we used the human breast cancer MDA-MB-231 cells (Figure 4C, 4D). We also examined the effect of BCI-121, a SMYD3 inhibitor [19], on the AKT activity. SW480 cells were treated with BCI-121 for 72 hours, followed by western blot analysis using anti-monomethyl AKT1 (K14) and anti-phospho AKT1 (Thr 308) antibodies (Figure 4E, 4F). As we expected, BCI-121 treatment significantly attenuated Lys 14 monomethylation and Thr 308 phosphorylation of AKT1 in a dose-dependent manner, further implying the importance of SMYD3-mediated methylation on AKT1 activation. This inhibitor effect was also validated in MDA-MB-231 cells (Figure 4G, 4H).

**Figure 5:**
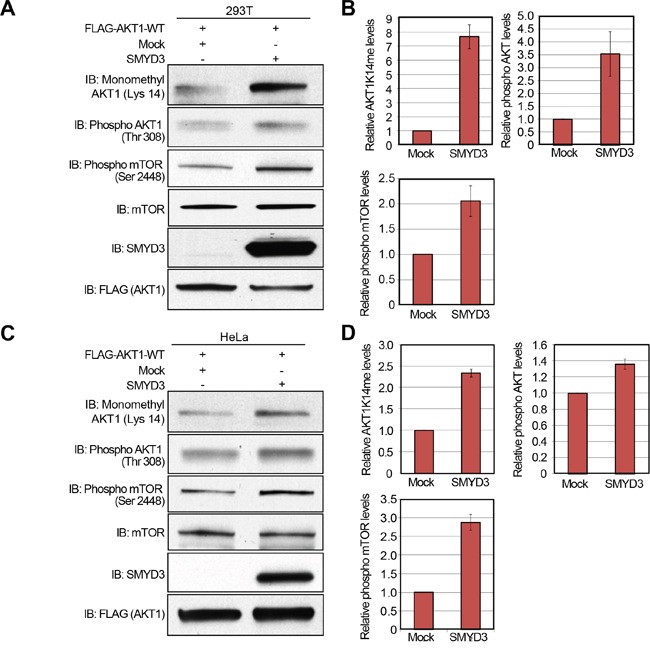
Overexpression of SMYD3 enhances the AKT pathway **A, C.** 293T cells (A) and HeLa cells (C) were transfected with Mock vector or SMYD3 expression vector (pcDNA3.1-SMYD3) together with wild-type FLAG-AKT1 expression vector (FLAG-AKT1-WT). After 48 hours of incubation, cells were treated with 100 ng/ml of EGF for 5 min, and then lysed with CelLytic™ M mammalian cell lysis/extraction reagent containing a protease inhibitor cocktail and a phosphatase cocktail. The samples were immunoblotted with anti-K14 monomethylated AKT1, anti-phospho AKT (Thr 308), anti-phospho mTOR (Ser 2448), anti-mTOR, anti-SMYD3 and anti-FLAG (internal control) antibodies. **B, D.** X-ray films examined in (A) and (C) were scanned with GS-800™ calibrated densitometer (Bio-Rad), and the intensity of monomethylated AKT1 (K14), phospho AKT (Thr 308) and phospho mTOR (Ser 2448) in 293T cells (B) and HeLa cells (D) was normalized by each total protein level.

Subsequently, to verify gain-of-function of SMYD3, we transfected wild-type FLAG-AKT1, and Mock or SMYD3 expression vector into 293T cells, and performed western blot analysis 48 hours after the transfection (Figure 5A). Quantification of western blot results revealed that SMYD3 overexpression enhances Lys 14 methylation and Thr 308 phopsphorylation of AKT1 as well as Ser 2448 phosphorylation of mTOR (Figure 5B). A similar result was observed when we used HeLa cells (Figure 5C, 5D), indicating that SMYD3-mediated methylation can enhance phosphorylation of Thr 308 on AKT1 and activate the AKT1 pathway *in vivo*.

### SMYD3-mediated lysine 14 methylation is required for the plasma membrane accumulation and AKT1-dependent growth promoting effects

**Figure 6:**
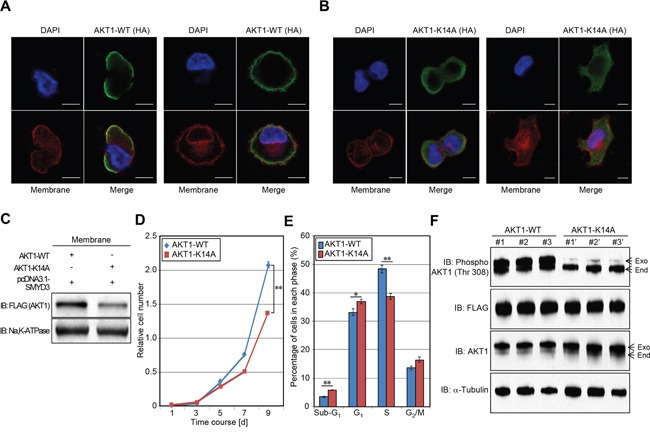
Methylation of AKT1 at lysine 14 promotes plasma membrane recruitment and proliferation of cancer cells **A-B.** HeLa cells were transfected with HA-tagged wild-type AKT1 (HA-AKT1-WT) (A) or K14A-substituted AKT1 (HA-AKT1-K14A) (B) in the presence of a SMYD3 expression vector (pcDNA3.1-SMYD3). After 48 hours of incubation, cells were trypsinized and re-seeded in 4-well chamber glass slides in a medium without FBS. After 24 hours of incubation, cells were treated with 100 ng/ml of EGF for 5 min, and then fixed with 4% paraformaldehyde. Fixed cells were stained with an anti-HA antibody (Alexa Fluor^®^ 488 [green]), Alexa Fluor^®^ 594 conjugated Wheat Germ Agglutinin (WGA) [red] and 4',6'-diamidine-2'-phenylindole dihydrochloride (DAPI [blue]). Scale bar, 10 μm. **C.** HeLa cells were transfected with HA-tagged wild-type AKT1 (HA-AKT1-WT) or K14A-substituted AKT1 (HA-AKT1-K14A) together with a SMYD3 expression vector (pcDNA3.1-SMYD3). After 48 hours of incubation, cells were treated with 100 ng/ml of EGF for 5 min, and then plasma membrane proteins were extracted using the Mem-PER™ plus membrane protein extraction kit (Thermo Fisher Scientific). Extracted proteins were bloated with an anti-FLAG antibody (Sigma-Aldrich), and an anti-Na, K-ATPase antibody was used as an internal control. **D.** Cell growth assays of SW480 cells expressing wild-type AKT1 or K14A substituted AKT1. Relative cell amount was measured by cell counting kit 8 (CCK8): results are the mean ±SD of three independent experiments. *P* values were calculated using Student's *t*-test (**, *P* < 0.01). **E.** Detailed cell cycle kinetics in SW480 cells expressing wild-type AKT of K14A substituted AKT1. Results are the mean ±SD of three independent experiments. *P* values were calculated using Student's *t*-test (*, *P* < 0.05; **, *P* < 0.01). **F.** Western blot analysis of SW480 cells expressing wild-type and K14A substituted AKT1. Samples were immnoblotted with anti-phospho AKT (Thr 308), anti-FLAG, anti-AKT and anti-α-Tubulin (internal control) antibodies.

Carpten *et al.* previously reported that glutamic acid 17 (Glu 17) substitution to lysine increased AKT1 presence at the plasma membrane and resulted in constitutive activation of AKT1 activity [6]. Given that the interaction between Lys 14 and Glu 17 seems to be critical for AKT1 activation [6], we examined the effect of SMYD3-meidiated Lys 14 methylation on the membrane accumulation of AKT1 (Figure 6A, 6B). HeLa cells were transfected with wild-type AKT1 or K14A-substituted AKT1 in the presence of the SMYD3 expression vector. After 48 hours of incubation, cells were re-seeded in 4-well chamber glass slides in a medium without fetal bovine serum (FBS). Subsequently, transfected cells were treated with Epidermal Growth Factor (EGF) for 5 min 24 h after incubation, and then, cells were fixed by 4% paraformaldehyde, followed by immunocytochemistry. Importantly, subcellular localization of wild-type AKT1 was merged with Alexa Fluor^®^ 594-conjugated Wheat Germ Agglutinin (WGA), a membrane marker, at the plasma membrane, which clearly showed the plasma membrane accumulation of AKT1. However, K14A-substituted AKT1 did not reveal such a clear accumulation pattern, implying that SMYD3-mediated Lys 14 methylation is likely to be important for the plasma membrane accumulation of AKT1 after the EGF stimulation. Moreover, we extracted plasma membrane proteins from HeLa cells transfected with wild-type AKT1 (AKT1-WT) or K14-substituted AKT1 (AKT1-K14A), and conducted western blot analysis. As shown in Figure 6C, amount of AKT1 protein at the plasma membrane was significantly decreased when Lys 14 was substituted to alanine, which further confirms that SMYD3-mediated Lys 14 methylation is critical for accumulation of AKT1 proteins at the plasma membrane.

To clarify the effect of SMYD3-mediated Lys 14 methylation on the AKT1 oncogenic activity, we generated SW480 stable transformants, which overexpress wild-type AKT1 (AKT1-WT) or Lys 14-substituted AKT1 (AKT1-K14A), and conducted a growth assay (Figure 6D). SW480 cells overexpressing K14A-substiuted AKT1 showed significantly lower growth rate than those overexpressing wild-type AKT1, which suggests that SMYD3-mediated Lys 14 methylation appears to be critical for the growth of SW480 cells. Consistently, according to detailed cell-cycle analysis by flow cytometry, SW480 cells overexpressing wild-type AKT1 showed significantly higher cell population in the S phase than those overexpressing K14A-substituted AKT1 (Figure 6E). Then we examined phosphorylation status of Thr 308 on AKT1 and confirmed significant attenuation of phosphorylation levels in SW480 cells overexpressing Lys 14-substituted AKT1 (Figure 6F). The data reveal that SMYD3-mediated Lys 14 methylation is critical for phosphorylation of Thr 308 on AKT1, which is required for the growth promoting effect.

## DISCUSSION

The AKT1 pathway plays a key role in many types of human cancer by regulating cell proliferation, differentiation, angiogenesis and apoptosis [25]. Although Phosphatidylinositide 3-kinase (PI3K)-dependent AKT1 phosphorylation by 3-Phosphoinositide Dependent Protein Kinase 1 (PDK1) and mTOR Complex 2 (mTORC2) has long been considered to be the primary mechanism accounting for AKT1 activation, these members are not sufficient enough to explain how AKT1 hyperactivation can occur in tumors with normal levels of PI3K/Phosphatase And Tensin Homolog (PTEN) activity [4].

In the present study, we have demonstrated that the protein lysine methyltransferase SMYD3 methylates Lys 14 in the PH domain of AKT1 both *in vitro* and *in vivo*, and that this methylation is pivotal for phosphorylation of Thr 308 that is known to be a marker for the AKT1 activation. In this regard, methylation of Lys 14 on AKT1 appears to promote the plasma membrane accumulation of AKT1 after the EGF stimulation. It is known that the binding of the PH domain to phosphoinositides activates AKT1. Thomas *et al.* previously reported that Lys 14 of AKT1 interacts with the lipid head group of PtdIns(3,4,5)P_3_ and PtsIns(3,4)P_2_ [23], which implies the importance of this lysine residue for the interaction between AKT1 and phosphoinositides. Since lysine methylation increases the basicity and hydrophobicity [27], the change of biochemical characteristics in the PH domain by SMYD3-mediated lysine methylation may enhance the binding affinity of AKT1 to phosphoinositides. Moreover, the rotatable bond between the amino nitrogen and the Cε atom of Lys 14 enables continuous changes of the rotamer of the added methyl group of Lys 14. Supplementary Figure S9 shows two different possible locations for the added methyl group. In a model in Supplementary Figure S9A, the methyl group is located at the maximum distance (4.05 Å), and in a model in Supplementary Figure S9B, it is at the minimum distance (1.93 Å). In either of the rotamers, the distance (≤ 4.05 Å) is enough to cause undesirable electrostatic interaction between the hydrophobic methyl group of Lys 14 and the polar carboxyl group of Glu 17. Importantly, Glu 17 occupies the phosphoinositide-binding pocket and forms a network of hydrogen bonds in the *apo* conformation [6, 22]. As Lys 14 methylation regulates the electrostatic attraction between Lys 14 and Glu 17, it can also affect the affinity of the PH domain of AKT1 to phosphoinositides. Furthermore, because Glu 17 shows the van der Waals contact with the activation loop, on which Thr 308 is located, SMYD3-mediated Lys 14 methylation would also influence the interaction between the β-hairpin loop in the PH domain and the activation loop through regulation of the electrostatic attraction between Lys 14 and Glu 17, which could enhance phosphorylation of Thr 308. On the basis of these results, Lys 14 of AKT1 is likely to be a key amino acid to determine AKT1 activity through the electrostatic attraction with Glu 17 and the interaction with phosphoinositides; SMYD3-mediated methylation of Lys 14 may trigger the constitutive activation of AKT1 in cancer cells.

In the recent years, the PH domain of AKT1 has been implicated as an important target for development of anti-cancer therapy [9, 28–30]. For instance, alkylphospholipids or ALPs are single-chain amphiphilic molecules that have been shown to reduce the levels of AKT1 membrane accumulation upon cell stimulation through binding to the PH domain [9, 31, 32]. If AKT1 loses the access or the affinity to the membrane, its phosphorylation level will be significantly reduced, and as a result, its kinase activity to transduce the growth signaling would be dramatically diminished. Indeed, Perifosine and Miltefosine are zwitterionic alkylphospholipids that hold promise as anticancer therapeutics [33–36]. Gradziel *et al.* recently demonstrated that Perifosine and Miltefosine bound to the PH domain through two discrete amphiphile binding sites on the domain as follows: (i) the cationic site that binds phosphoinositides and some alkylphospholipids and (ii) a second site that is occupied by only the alkylphospholipids [9]. In their model, Glu 17 interacts with the cationic moieties of Perifosine and Miltefosine and stabilizes their binding to the PH domain. Taken together, these results clearly indicate that SMYD3-mediated Lys 14 methylation on AKT1 must be a good target for anti-cancer treatment. In fact, overexpression of SMYD3 is observed in a wide variety of cancer tissues [10–12] (Supplementary Figures S2 and S3), and inactivation of SMYD3 results in growth suppression of various types of cancer cells (Supplementary Figure S10) [10, 12]. Besides, we confirmed that the SMYD3 inhibitor BCI-121 significantly diminished Thr 308 phosphorylation levels of AKT1; this inhibitor effectively repressed the growth of cancer cells overexpressing SMYD3 [19], further supporting the potential of SMYD3 as a target of anti-cancer drug development.

In summary, our findings unveiled the novel mechanism of constitutive hyperphosphorylation on AKT1 in cancer cells, mediated by SMYD3-dependent methylation. The inhibition of this methylation pathway may be a promising strategy to develop anti-cancer drugs.

## MATERIALS AND METHODS

### Cell culture

Human colon cancer cell line SW480, human metastatic breast cancer cell line MDA-MB-231, human embryonic kidney cell line 293T and human cervical cancer cell line HeLa were from American Type Culture Collection (ATCC) in 2003 and 2013, and tested and authenticated by DNA profiling for polymorphic short tandem repeat (STR) markers (Supplementary Table S1). All cell lines were grown in monolayers in appropriate media: Dulbecco's modified Eagle's medium (D-MEM) for 293T cells; Eagle's Minimum Essential Medium (E-MEM) for HeLa cells; Leibovitz's L-15 for SW480 and MDA-MB-231 cells supplemented with 10% fetal bovine serum and 1% antibiotic/antimycotic solution (Sigma-Aldrich). All cells were maintained at 37°C in humid air with 5% CO_2_ condition (293T and HeLa) or without CO_2_ (SW480 and MDA-MB-231). Cells were transfected with FuGENE^®^ 6 or FuGENE^®^ HD (Promega) transfection reagent according to manufacturer's protocols [10, 12].

### Antibodies

The following primary antibodies were used; anti-FLAG (rabbit, F7425; Sigma-Aldrich; dilution used in WB: 1:1000), anti-HA (rat, #11867423001; Roche Applied Science; dilution used in ICC: 1:1000), anti-SMYD3 (rabbit, D2Q4V; Cell Signaling Technology; dilution used in WB: 1:1000), anti-AKT (rabbit, C67E7; Cell Signaling Technology; dilution used in WB: 1:1000), anti-phospho-AKT (Thr 308) (rabbit, D25E6; Cell Signaling Technology; dilution used in WB: 1:500), anti-α-Tubulin (mouse, DM1A; CALBIOCHEM; dilution used in WB: 1:1000), anti-mTOR (rabbit, 7C10; Cell Signaling Technology; dilution used in WB: 1:1000), anti-phospho mTOR (Ser 2448) (rabbit, D9C2; Cell Signaling Technology; dilution used in WB: 1:500) and anti-Na, K-ATPase (rabbit, #3010; Cell Signaling Technology; dilution used in WB: 1:1000). An anti-K14 monomethylated AKT1 antibody (AnaSpec/Eurogentec; dilution used in WB: 1:1000) was produced in rabbit immunized with a synthetic peptide.

### *In vitro* methyltransferase assay

For the *in vitro* methyltransferase assay, recombinant AKT1 (P2999, Thermo Fisher Scientific) was incubated with recombinant SMYD3 enzyme using 2 μCi S-adenosyl-L-[methyl-^3^H]-methionine (SAM; PerkinElmer) as the methyl donor in a mixture of 10 μl of methylase activity buffer (50 mM Tris-HCl at pH8.8, 10 mM dithiothreitol (DTT) and 10 mM MgCl_2_), for 2 hours at 30°C [37–46]. Proteins were resolved on a Mini-PROTEAN^®^ TGX™ Precast gel (Any kD; Bio-Rad) and visualized by fluorography using EN^3^HANCE™ Spray Surface Autoradiography Enhancer (PerkinElmer). Loading proteins were visualized by MemCode™ Reversible Stain (Thermo Fisher Scientific).

### Mass spectrometry

AKT1 samples reacted with bovine serum albumin (BSA) or SMYD3 *in vitro* and immunoprecipitated AKT1 samples from 293T cells and HeLa cells were separated on SDS-PAGE and stained with Simply Blue Safe Stain (Thermo Fisher Scientific). The AKT1 bands were excised and digested in gel with trypsin or endoproteinase Asp-N. Then digest was analyzed by nano liquid chromatography–tandem mass spectrometry (LC-MS/MS) using Q Exactive mass spectrometer (Thermo Fisher Scientific). The peptides were separated using nano ESI spray column (75 μm [ID] × 100 mm [L], NTCC analytical column C18, 3 μm, Nikkyo Technos) with a linear gradient of 0%–35% buffer B (100% acetonitrile and 0.1% formic acid) at a flow rate of 300 nL/min over 10 min (Easy nLC; Thermo Fisher Scientific). The mass spectrometer was operated in the positive-ion mode, and the MS and MS/MS spectra were acquired in a data-dependent TOP10 method. The MS/MS spectra were searched against the in-house database using local MASCOT server (version 2.5; Matrix Sciences). For the quantitative analysis *in vivo* methylation, AKT1 peptides were monitored using targeted MS/MS method.

### Western blot

Samples were prepared from the cells lysed with CelLytic™ M mammalian cell lysis/extraction reagent (Sigma-Aldrich) [39] containing a complete protease inhibitor cocktail (Roche Applied Science) and a phosphatase inhibitor cocktail (Roche Applied Science), and whole cell lysates or IP products were transferred to nitrocellulose membrane. Protein bands were detected by incubating with horseradish peroxidase-conjugated antibodies (GE Healthcare) and visualizing with Enhanced Chemiluminescence (GE Healthcare). We declare that our blots were evenly exposed in each membrane and that the blots are not clopped to the bands.

### Immunoprecipitation

293T cells were transfected with wild-type AKT1 expression vector (pcDNA3.1-FLAG-HA-AKT1) or mutant-type AKT1 expression vector (pcDNA3.1-FLAG-HA-AKT1-K14A, K14R, K30A, K39A, K14A/K30A, K14A/K39A, K30A/K39A, K14A/K30A/K39A) in the presence of pcDNA3.1-SMYD3 expression vector. After 48 hours of incubation, cells were treated with 100 ng/ml of EGF, and then lysed with CelLytic™ M mammalian cell lysis/extraction reagent (Sigma-Aldrich) containing a complete protease inhibitor cocktail (Roche Applied Science) and a phosphatase inhibitor cocktail (Roche Applied Science). Three-hundred micrograms of whole-cell extract were incubated with anti-FLAG^®^ M2 affinity gel (Sigma-Aldrich) overnight. After the beads had been washed 3 times in 1 ml of PBS buffer, proteins that bound to the beads were eluted by elution buffer (100 μg/ml 3X FLAG^®^ Peptide (Sigma-Aldrich) in PBS) containing a complete protease inhibitor cocktail (Roche Applied Science). Eluted samples were boiled with Lane Marker Reducing Sample Buffer (Thermo Fisher Scientific), followed by western blot analysis.

### Enzyme-linked immunosorbent assay (ELISA)

The ELISA test carried out was an indirect ELISA. A constant amount of antigen has been coated into the wells of the ELISA plate (10 μg of peptide/well), and tested with different dilutions of the serum or antibody. The development is done colorimetric, using a secondary HRP-conjugated antibody, and o-phenylenediamine as chromogenic substrate. The optical density of the chromogenic substrate is measured at 492 nm OD.

### siRNA transfection

Small interfering RNA (siRNA) oligonucleotide duplexes were purchased from Sigma-Aldrich for targeting the human *SMYD3* transcript. siEGFP was used as a control siRNA [47–52]. The siRNA sequences are described in Supplementary Table S2. siRNA duplexes (100 nM final concentration) were transfected into SW480 and MDA-MB-231 cells with Lipofectamine^®^ RNAiMax Reagent (Thermo Fisher Scientific).

### BGI-121 treatment

BGI-121, a SMYD3 specific inhibitor, was developed by Dr. Cristiano Simone group [19]. SW480 cells and MDA-MB-231 cells were treated with BGI-121 at the concentration of 0, 10 and 50 μM. After 72 hours of incubation, cells were treated with 100 ng/ml of EGFR (SW480) and 100 ng/ml neuregulin 1 (NRG1, MDA-MB-231) for 5 min, and then lysed with CelLytic™ M mammalian cell lysis/extraction reagent with a complete protease inhibitor cocktail (Roche Applied Science) and a phosphatase inhibitor cocktail (Roche Applied Science). Cell lysates were separated by SDS-PAGE, followed by western blot analysis using anti-K14 monomethylated AKT1 antibody, anti-phospho AKT (Thr 308, Cell Signaling Technology), anti-AKT (Cell Signaling Technology) and anti-α-Tubulin (CALBIOCHEM).

### Immunocytochemistry

HeLa cells were plated in two 10 cm dishes at the concentration of 1 × 10^6^ cells/dish and after 24 hours of incubation, cells were transfected with wild-type AKT1 expression vector (pcDNA3.1-FLAG-HA-AKT1) or Lys 14-substituted AKT1 (pcDNA3.1-FLAG-HA-AKT1-K14A) using Fugene^®^ HD transfection reagent (Promega). After 48 hours of transfection, cells were trypsinized and re-seeded in 4 well chamber glass slides at the concentration of 2 × 10^4^ cells/well in a cell culture medium without FBS. After 24 hours of incubation, cells were treated with 100 ng/ml of EGF for 5 min, and then washed 2 times with phosphate-buffered saline (PBS). Following suctioning of PBS, 4% paraformaldehyde was added to each well for 30 min at 4°C to fix the cells. Subsequently, cells were washed with PBS three times for 5 min each time at room temperature. 0.1% Triton X-100 was added for 3 min at room temperature to permeabilize the cells and samples were washed with PBS three times for 5 min each time. Then cells were blocked with 3% BSA for 1 hour at room temperature and incubated with anti-HA antibody (Roche Applied Science, dilution: 1: 1000) in 3% BSA solution at 4°C overnight. Subsequently, cells were washed 3 times with PBS, and incubated with secondary antibody (Alexa Fluor^®^ 488 conjugated anti-rat, Thermo Fisher Scientific, dilution: 1:1000) and 5 μg/ml of Alexa Fluor^®^ 594 conjugated Wheat Germ Agglutinin (WGA) for 1 hour at room temperature with gentle shaking. Following this, cells were washed 4 times with PBS and mounting medium with 4',6'-diamidine-2'-phenylindole dihydrochloride (DAPI, VECTASHIELD^®^, Vector Laboratories) was added on each well [53]. The wells were finally covered with a glass slide. Leica confocal microscopy (SP5 Tandem Scanner Spectral 2-Photon Confocal) was used for observation of stained cells [54].

### Generation of AKT1-overexpressing cells

SW480 cells were cultured in Leibovitz's L-15 medium with 10% FBS for 24 hours and transfected with FLAG-HA-AKT1-WT expression vector or FLAG-HA-AKT1-K14A expression vector using FuGENE^®^ HD transfection reagent (Promega). After 48 hours of incubation, cells were trypsinized and re-seeded in 10 cm dishes in a medium containing 1.0 mg/ml of Geneticin^®^ (Thermo Fisher Scientific). Cells were cultured in a medium containing 1.0 mg/ml of Geneticin^®^ for 15 days, and then colonies were picked up using the sterile cloning cylinders. To examine expression levels of AKT1 and phosphorylated AKT1 at Thr 308, cells were lysed with CelLytic™ M mammalian cell lysis/extraction reagent (Sigma-Aldrich) containing a complete protease inhibitor cocktail (Roche Applied Science) and a phosphatase inhibitor cocktail (Roche Applied Science). Lysed samples were immunoblotted with anti-phospho AKT (Thr 308, Cell Signaling Technology), anti-FLAG (Sigma-Aldrich), anti-AKT (Cell Signaling Technology) and anti-α-Tubulin (CALBIOCHEM) antibodies.

### Coupled cell cycle and cell proliferation assay

The 5'-bromo-2'-deoxyuridine (BrdU) flow kit (BD Biosciences) was used to determine the cell cycle kinetics and to measure the incorporation of BrdU into DNA of proliferating cells [37, 55–58]. The assay was performed according to the manufacturer's protocol. Briefly, SW480 cells overexpressing wild-type AKT1 (WT) or Lys 14-substituted AKT1 (AKT1-K14A) were cultured in the presence of 10 μM of BrdU for 30 min at 37°C. Then both floating and adherent cells were pooled from triplicate wells per treatment point, fixed in a solution containing paraformaldehyde and the detergent saponin, and incubated for 1 hour with DNase at 37°C (30 μg per sample). Fluorescein isothiocyanate (FITC)-conjugated anti-BrdU antibody (1:50 dilution in Wash buffer; BD Biosciences) was added and incubation continued for 20 min at room temperature. Cells were washed in Wash buffer and total DNA was stained with 7-amino-actinomycin D (7-AAD; 20 μL per sample), followed by flow cytometric analysis using BD™ LSR II (BD Biosciences) and FlowJo software.

### Extraction of plasma membrane proteins

HeLa cells were plated in two 10 cm dishes at the concentration of 1 × 10^6^ cells/dish and after 24 hours of incubation, cells were transfected with wild-type AKT1 expression vector (pcDNA3.1-FLAG-HA-AKT1) or Lys 14-substituted AKT1 (pcDNA3.1-FLAG-HA-AKT1-K14A) using Fugene^®^ HD transfection reagent (Promega). After 48 hours of incubation, cells were treated with 100 ng/ml of EGF for 5 min, and then harvested by cell scraper. The Mem-PER™ plus membrane protein extraction kit (Thermo Fisher Scientific) was used for the enrichment of plasma membrane proteins from harvested cells. The extraction was performed according to the manufacturer's protocol.

### Statistical analysis

Statistical analyses were performed using Student's *t*-test and results are the mean ±standard deviation (SD) of three independent experiments. Statistical significance was assumed when *P* < 0.05. No samples or data were excluded from the analysis. No power calculation was performed to determine the number of samples required to achieve statistical significance.

## SUPPLEMENTARY FIGURES AND TABLES



## References

[1] Altomare DA, Testa JR (2005). Perturbations of the AKT signaling pathway in human cancer. Oncogene.

[2] Kumar A, Rajendran V, Sethumadhavan R, Purohit R (2013). AKT kinase pathway: a leading target in cancer research. ScientificWorldJournal.

[3] Kumar A, Purohit R (2013). Cancer associated E17K mutation causes rapid conformational drift in AKT1 pleckstrin homology (PH) domain. PLoS One.

[4] Chan CH, Jo U, Kohrman A, Rezaeian AH, Chou PC, Logothetis C, Lin HK (2014). Posttranslational regulation of Akt in human cancer. Cell Biosci.

[5] Cheung M, Testa JR (2013). Diverse mechanisms of AKT pathway activation in human malignancy. Curr Cancer Drug Targets.

[6] Carpten JD, Faber AL, Horn C, Donoho GP, Briggs SL, Robbins CM, Hostetter G, Boguslawski S, Moses TY, Savage S, Uhlik M, Lin A, Du J (2007). A transforming mutation in the pleckstrin homology domain of AKT1 in cancer. Nature.

[7] Parikh C, Janakiraman V, Wu WI, Foo CK, Kljavin NM, Chaudhuri S, Stawiski E, Lee B, Lin J, Li H, Lorenzo MN, Yuan W, Guillory J (2012). Disruption of PH-kinase domain interactions leads to oncogenic activation of AKT in human cancers. Proc Natl Acad Sci U S A.

[8] Landgraf KE, Pilling C, Falke JJ (2008). Molecular mechanism of an oncogenic mutation that alters membrane targeting: Glu17Lys modifies the PIP lipid specificity of the AKT1 PH domain. Biochemistry.

[9] Gradziel CS, Wang Y, Stec B, Redfield AG, Roberts MF (2014). Cytotoxic amphiphiles and phosphoinositides bind to two discrete sites on the Akt1 PH domain. Biochemistry.

[10] Hamamoto R, Furukawa Y, Morita M, Iimura Y, Silva FP, Li M, Yagyu R, Nakamura Y (2004). SMYD3 encodes a histone methyltransferase involved in the proliferation of cancer cells. Nat Cell Biol.

[11] Hamamoto R, Saloura V, Nakamura Y (2015). Critical roles of non-histone protein lysine methylation in human tumorigenesis. Nat Rev Cancer.

[12] Hamamoto R, Silva FP, Tsuge M, Nishidate T, Katagiri T, Nakamura Y, Furukawa Y (2006). Enhanced SMYD3 expression is essential for the growth of breast cancer cells. Cancer Sci.

[13] Tsuge M, Hamamoto R, Silva FP, Ohnishi Y, Chayama K, Kamatani N, Furukawa Y, Nakamura Y (2005). A variable number of tandem repeats polymorphism in an E2F-1 binding element in the 5' flanking region of SMYD3 is a risk factor for human cancers. Nat Genet.

[14] Silva FP, Hamamoto R, Kunizaki M, Tsuge M, Nakamura Y, Furukawa Y (2008). Enhanced methyltransferase activity of SMYD3 by the cleavage of its N-terminal region in human cancer cells. Oncogene.

[15] Kunizaki M, Hamamoto R, Silva FP, Yamaguchi K, Nagayasu T, Shibuya M, Nakamura Y, Furukawa Y (2007). The lysine 831 of vascular endothelial growth factor receptor 1 is a novel target of methylation by SMYD3. Cancer Res.

[16] Mazur PK, Reynoird N, Khatri P, Jansen PW, Wilkinson AW, Liu S, Barbash O, Van Aller GS, Huddleston M, Dhanak D, Tummino PJ, Kruger RG, Garcia BA (2014). SMYD3 links lysine methylation of MAP3K2 to Ras-driven cancer. Nature.

[17] Liu Y, Liu H, Luo X, Deng J, Pan Y, Liang H (2015). Overexpression of SMYD3 and matrix metalloproteinase-9 are associated with poor prognosis of patients with gastric cancer. Tumour Biol.

[18] Liu Y, Luo X, Deng J, Pan Y, Zhang L, Liang H (2015). SMYD3 overexpression was a risk factor in the biological behavior and prognosis of gastric carcinoma. Tumour Biol.

[19] Peserico A, Germani A, Sanese P, Barbosa AJ, Di Virgilio V, Fittipaldi R, Fabini E, Bertucci C, Varchi G, Moyer MP, Caretti G, Del Rio A, Simone C (2015). A SMYD3 Small-Molecule Inhibitor Impairing Cancer Cell Growth. J Cell Physiol.

[20] Sponziello M, Durante C, Boichard A, Dima M, Puppin C, Verrienti A, Tamburrano G, Di Rocco G, Redler A, Lacroix L, Bidart JM, Schlumberger M, Damante G (2014). Epigenetic-related gene expression profile in medullary thyroid cancer revealed the overexpression of the histone methyltransferases EZH2 and SMYD3 in aggressive tumours. Mol Cell Endocrinol.

[21] Vieira FQ, Costa-Pinheiro P, Almeida-Rios D, Graca I, Monteiro-Reis S, Simoes-Sousa S, Carneiro I, Sousa EJ, Godinho MI, Baltazar F, Henrique R, Jeronimo C (2015). SMYD3 contributes to a more aggressive phenotype of prostate cancer and targets Cyclin D2 through H4K20me3. Oncotarget.

[22] Milburn CC, Deak M, Kelly SM, Price NC, Alessi DR, Van Aalten DM (2003). Binding of phosphatidylinositol 3,4,5-trisphosphate to the pleckstrin homology domain of protein kinase B induces a conformational change. Biochem J.

[23] Thomas CC, Deak M, Alessi DR, van Aalten DM (2002). High-resolution structure of the pleckstrin homology domain of protein kinase b/akt bound to phosphatidylinositol (3,4,5)-trisphosphate. Curr Biol.

[24] Vincent EE, Elder DJ, Thomas EC, Phillips L, Morgan C, Pawade J, Sohail M, May MT, Hetzel MR, Tavare JM (2011). Akt phosphorylation on Thr308 but not on Ser473 correlates with Akt protein kinase activity in human non-small cell lung cancer. Br J Cancer.

[25] Gallay N, Dos Santos C, Cuzin L, Bousquet M, Simmonet Gouy V, Chaussade C, Attal M, Payrastre B, Demur C, Recher C (2009). The level of AKT phosphorylation on threonine 308 but not on serine 473 is associated with high-risk cytogenetics and predicts poor overall survival in acute myeloid leukaemia. Leukemia.

[26] Bellacosa A, Kumar CC, Di Cristofano A, Testa JR (2005). Activation of AKT kinases in cancer: implications for therapeutic targeting. Adv Cancer Res.

[27] Rice JC, Nishioka K, Sarma K, Steward R, Reinberg D, Allis CD (2002). Mitoticspecific methylation of histone H4 Lys 20 follows increased PR-Set7 expression and its localization to mitotic chromosomes. Genes Dev.

[28] Harrison C (2011). Anticancer drugs: blocking phospholipid-protein interactions. Nat Rev Drug Discov.

[29] Mahadevan D, Powis G, Mash EA, George B, Gokhale VM, Zhang S, Shakalya K, Du-Cuny L, Berggren M, Ali MA, Jana U, Ihle N, Moses S (2008). Discovery of a novel class of AKT pleckstrin homology domain inhibitors. Mol Cancer Ther.

[30] Joh EH, Hollenbaugh JA, Kim B, Kim DH (2012). Pleckstrin homology domain of Akt kinase: a proof of principle for highly specific and effective non-enzymatic anticancer target. PLoS One.

[31] Gills JJ, Dennis PA (2009). Perifosine: update on a novel Akt inhibitor. Curr Oncol Rep.

[32] van Blitterswijk WJ, Verheij M (2013). Anticancer mechanisms and clinical application of alkylphospholipids. Biochim Biophys Acta.

[33] Kondapaka SB, Singh SS, Dasmahapatra GP, Sausville EA, Roy KK (2003). Perifosine, a novel alkylphospholipid, inhibits protein kinase B activation. Mol Cancer Ther.

[34] Pachioni Jde A, Magalhaes JG, Lima EJ, Bueno Lde M, Barbosa JF, de Sa MM, Rangel-Yagui CO (2013). Alkylphospholipids - a promising class of chemotherapeutic agents with a broad pharmacological spectrum. J Pharm Pharm Sci.

[35] Guidetti A, Carlo-Stella C, Locatelli SL, Malorni W, Mortarini R, Viviani S, Russo D, Marchiano A, Sorasio R, Dodero A, Farina L, Giordano L, Di Nicola M (2014). Phase II study of perifosine and sorafenib dual-targeted therapy in patients with relapsed or refractory lymphoproliferative diseases. Clin Cancer Res.

[36] Chakrabandhu K, Huault S, Hueber AO (2008). Distinctive molecular signaling in triple-negative breast cancer cell death triggered by hexadecylphosphocholine (miltefosine). FEBS Lett.

[37] Cho HS, Hayami S, Toyokawa G, Maejima K, Yamane Y, Suzuki T, Dohmae N, Kogure M, Kang D, Neal DE, Ponder BA, Yamaue H, Nakamura Y (2012). RB1 methylation by SMYD2 enhances cell cycle progression through an increase of RB1 phosphorylation. Neoplasia.

[38] Takawa M, Cho HS, Hayami S, Toyokawa G, Kogure M, Yamane Y, Iwai Y, Maejima K, Ueda K, Masuda A, Dohmae N, Field HI, Tsunoda T (2012). Histone Lysine Methyltransferase SETD8 Promotes Carcinogenesis by Deregulating PCNA Expression. Cancer Res.

[39] Cho HS, Shimazu T, Toyokawa G, Daigo Y, Maehara Y, Hayami S, Ito A, Masuda K, Ikawa N, Field HI, Tsuchiya E, Ohnuma S, Ponder BA (2012). Enhanced HSP70 lysine methylation promotes proliferation of cancer cells through activation of Aurora kinase B. Nat Commun.

[40] Cho HS, Suzuki T, Dohmae N, Hayami S, Unoki M, Yoshimatsu M, Toyokawa G, Takawa M, Chen T, Kurash JK, Field HI, Ponder BA, Nakamura Y (2011). Demethylation of RB regulator MYPT1 by histone demethylase LSD1 promotes cell cycle progression in cancer cells. Cancer Res.

[41] Sone K, Piao L, Nakakido M, Ueda K, Jenuwein T, Nakamura Y, Hamamoto R (2014). Critical role of lysine 134 methylation on histone H2AX for gamma-H2AX production and DNA repair. Nat Commun.

[42] Nakakido M, Deng Z, Suzuki T, Dohmae N, Nakamura Y, Hamamoto R (2015). Dysregulation of AKT Pathway by SMYD2-Mediated Lysine Methylation on PTEN. Neoplasia.

[43] Hamamoto R, Toyokawa G, Nakakido M, Ueda K, Nakamura Y (2014). SMYD2-dependent HSP90 methylation promotes cancer cell proliferation by regulating the chaperone complex formation. Cancer Lett.

[44] Kogure M, Takawa M, Saloura V, Sone K, Piao L, Ueda K, Ibrahim R, Tsunoda T, Sugiyama M, Atomi Y, Nakamura Y, Hamamoto R (2013). The oncogenic polycomb histone methyltransferase EZH2 methylates lysine 120 on histone H2B and competes ubiquitination. Neoplasia.

[45] Nakakido M, Deng Z, Suzuki T, Dohmae N, Nakamura Y, Hamamoto R (2015). PRMT6 increases cytoplasmic localization of p21CDKN1A in cancer cells through arginine methylation and makes more resistant to cytotoxic agents. Oncotarget.

[46] Piao L, Suzuki T, Dohmae N, Nakamura Y, Hamamoto R (2015). SUV39H2 methylates and stabilizes LSD1 by inhibiting polyubiquitination in human cancer cells. Oncotarget.

[47] Yoshimatsu M, Toyokawa G, Hayami S, Unoki M, Tsunoda T, Field HI, Kelly JD, Neal DE, Maehara Y, Ponder BA, Nakamura Y, Hamamoto R (2011). Dysregulation of PRMT1 and PRMT6, Type I arginine methyltransferases, is involved in various types of human cancers. Int J Cancer.

[48] Takawa M, Masuda K, Kunizaki M, Daigo Y, Takagi K, Iwai Y, Cho HS, Toyokawa G, Yamane Y, Maejima K, Field HI, Kobayashi T, Akasu T (2011). Validation of the histone methyltransferase EZH2 as a therapeutic target for various types of human cancer and as a prognostic marker. Cancer Sci.

[49] Toyokawa G, Masuda K, Daigo Y, Cho HS, Yoshimatsu M, Takawa M, Hayami S, Maejima K, Chino M, Field HI, Neal DE, Tsuchiya E, Ponder BA (2011). Minichromosome Maintenance Protein 7 is a potential therapeutic target in human cancer and a novel prognostic marker of non-small cell lung cancer. Mol Cancer.

[50] Kogure M, Takawa M, Cho HS, Toyokawa G, Hayashi K, Tsunoda T, Kobayashi T, Daigo Y, Sugiyama M, Atomi Y, Nakamura Y, Hamamoto R (2013). Deregulation of the histone demethylase JMJD2A is involved in human carcinogenesis through regulation of the G(1)/S transition. Cancer Lett.

[51] Toyokawa G, Cho HS, Iwai Y, Yoshimatsu M, Takawa M, Hayami S, Maejima K, Shimizu N, Tanaka H, Tsunoda T, Field HI, Kelly JD, Neal DE (2011). The histone demethylase JMJD2B plays an essential role in human carcinogenesis through positive regulation of cyclin-dependent kinase 6. Cancer Prev Res (Phila).

[52] Kang D, Cho HS, Toyokawa G, Kogure M, Yamane Y, Iwai Y, Hayami S, Tsunoda T, Field HI, Matsuda K, Neal DE, Ponder BA, Maehara Y (2013). The histone methyltransferase Wolf-Hirschhorn syndrome candidate 1-like 1 (WHSC1L1) is involved in human carcinogenesis. Genes Chromosomes Cancer.

[53] Saloura V, Cho HS, Kyiotani K, Alachkar H, Zuo Z, Nakakido M, Tsunoda T, Seiwert T, Lingen M, Licht J, Nakamura Y, Hamamoto R (2015). WHSC1 Promotes Oncogenesis through Regulation of NIMA-related-kinase-7 in Squamous Cell Carcinoma of the Head and Neck. Mol Cancer Res.

[54] Piao L, Kang D, Suzuki T, Masuda A, Dohmae N, Nakamura Y, Hamamoto R (2014). The histone methyltransferase SMYD2 methylates PARP1 and promotes poly(ADPribosyl) ation activity in cancer cells. Neoplasia.

[55] Hayami S, Kelly JD, Cho HS, Yoshimatsu M, Unoki M, Tsunoda T, Field HI, Neal DE, Yamaue H, Ponder BA, Nakamura Y, Hamamoto R (2011). Overexpression of LSD1 contributes to human carcinogenesis through chromatin regulation in various cancers. Int J Cancer.

[56] Hayami S, Yoshimatsu M, Veerakumarasivam A, Unoki M, Iwai Y, Tsunoda T, Field HI, Kelly JD, Neal DE, Yamaue H, Ponder BA, Nakamura Y, Hamamoto R (2010). Overexpression of the JmjC histone demethylase KDM5B in human carcinogenesis: involvement in the proliferation of cancer cells through the E2F/RB pathway. Mol Cancer.

[57] Toyokawa G, Cho HS, Masuda K, Yamane Y, Yoshimatsu M, Hayami S, Takawa M, Iwai Y, Daigo Y, Tsuchiya E, Tsunoda T, Field HI, Kelly JD (2011). Histone Lysine Methyltransferase Wolf-Hirschhorn Syndrome Candidate 1 Is Involved in Human Carcinogenesis through Regulation of the Wnt Pathway. Neoplasia.

[58] Cho HS, Toyokawa G, Daigo Y, Hayami S, Masuda K, Ikawa N, Yamane Y, Maejima K, Tsunoda T, Field HI, Kelly JD, Neal DE, Ponder BA (2012). The JmjC domain-containing histone demethylase KDM3A is a positive regulator of the G1/S transition in cancer cells via transcriptional regulation of the HOXA1 gene. Int J Cancer.

[59] Ashwell MA, Lapierre JM, Brassard C, Bresciano K, Bull C, Cornell-Kennon S, Eathiraj S, France DS, Hall T, Hill J, Kelleher E, Khanapurkar S, Kizer D (2012). Discovery and optimization of a series of 3-(3-phenyl-3H-imidazo[4,5-b]pyridin-2-yl)pyridin-2-amines: orally bioavailable, selective, and potent ATP-independent Akt inhibitors. J Med Chem.

