# Supplementary material for: SMYD3-mediated lysine methylation in the PH domain is critical for activation of AKT1
Source: Oncotarget. 2016 Sep 8;7(46):75023–37. doi: 10.18632/oncotarget.11898 (PMC5342720; doi:10.18632/oncotarget.11898)
Supplement: Supplementary file 1 [file oncotarget-07-75023-s001.pdf]

SMYD3-mediated lysine methylation in the PH domain is critical for activation of AKT1

SUPPLEMENTARY FIGURES AND TABLES

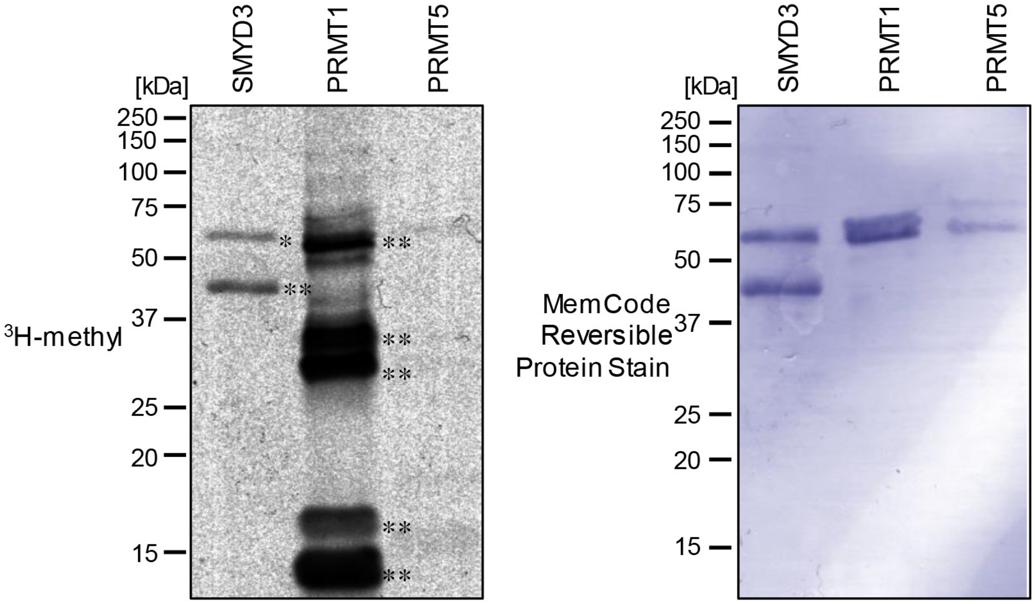

Supplementary Figure S1: SMYD3 methylates AKT1 *in vitro*. Recombinant AKT1 protein was incubated with SMYD3, PRMT1 and PRMT5 in the presence of S-adenosyl-L-[methyl-^{3}H]-methionine, and methylation signal was detected by autoradiography (left panel). Amounts of loading proteins were evaluated by staining with MemCode™ Reversible Protein Stain (right panel). *, AKT1 methylation band. **, automethylation band.

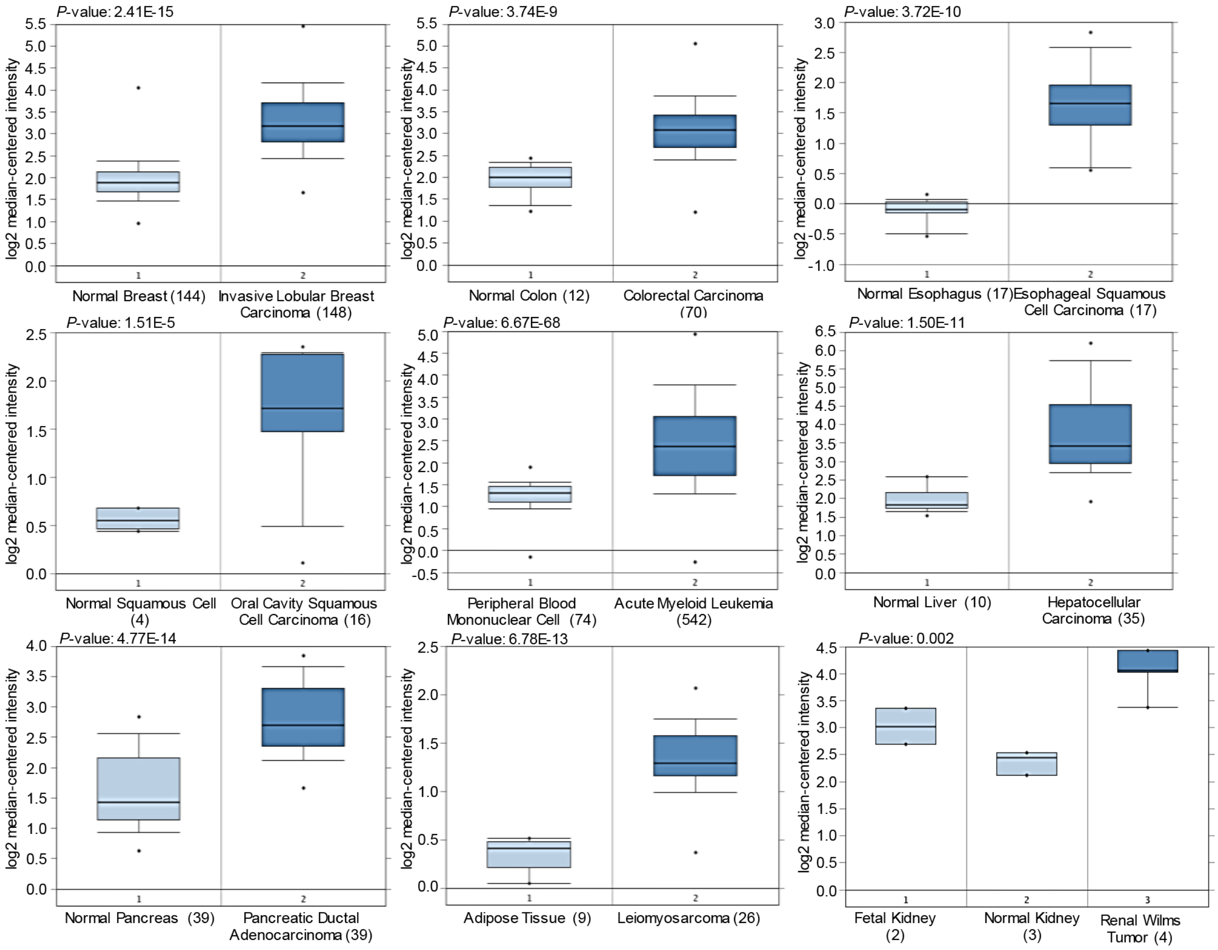

Supplementary Figure S2: SMYD3 is overexpressed in various types of cancer. Gene expression data in Oncomine was analyzed. The thick bars in the boxes are average expression levels and the boxes represent 95% of the samples. The error bars are above or below the boxes, and the range of expression levels is enclosed by two dots.

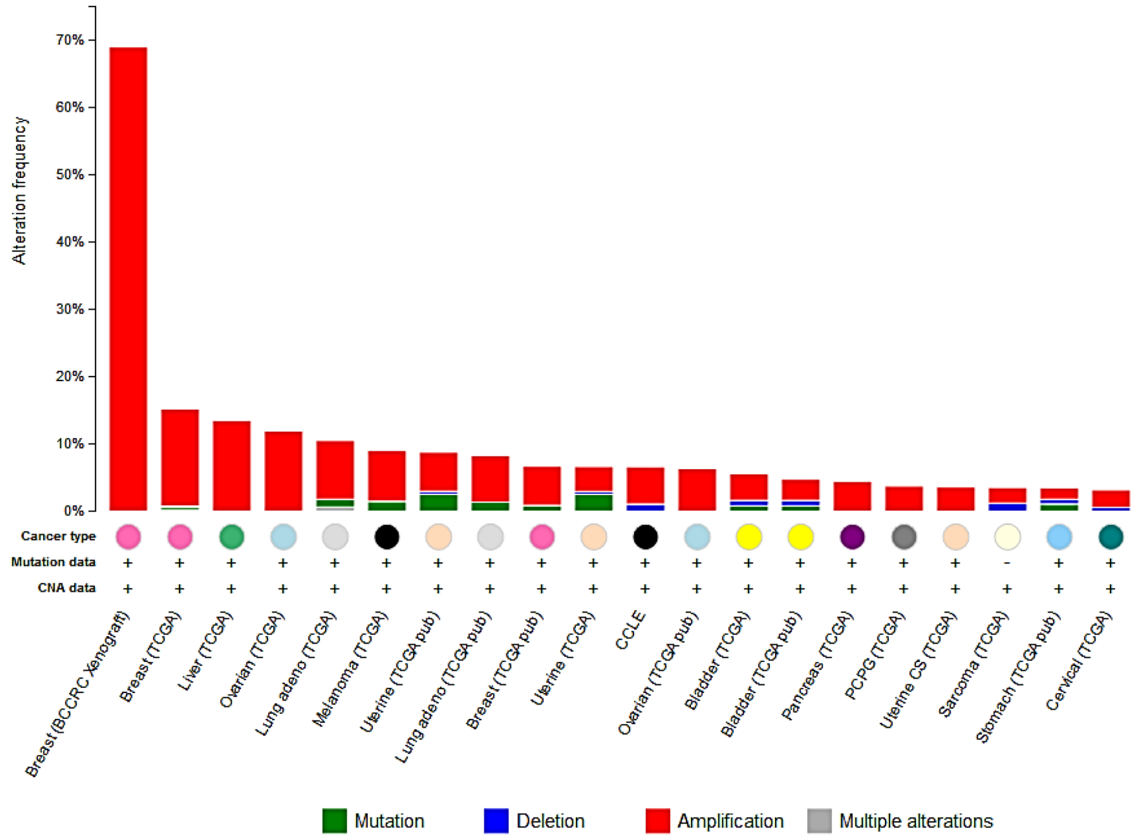

Supplementary Figure S3: Cross-cancer alteration summary for SMYD3. The data were obtained from the TCGA database (http://www.cbioportal.org/public-portal/).

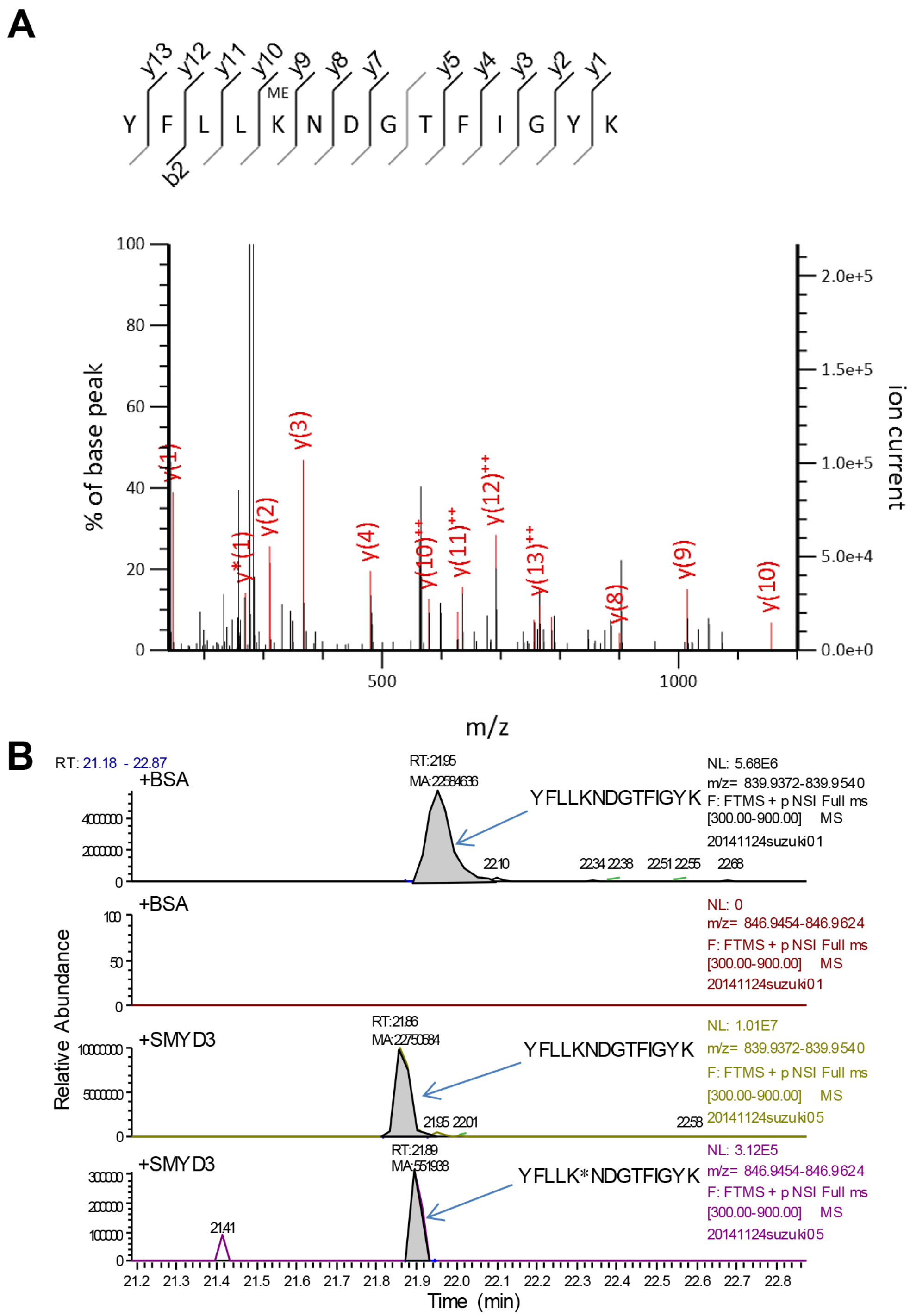

Supplementary Figure S4: Lysine 30 on AKT1 is methylated by SMYD3. A. The MS-MS spectrum corresponding to the monomethylated AKT1 26-39 peptide. The 14 Da increase of the lysine 30 residue was observed. **B.** MS chromatograms of unmodified and monomethylated AKT1 26-39 peptides. Score and Expect show Mascot Ion Score and Expectation value in Mascot Database search results, respectively.

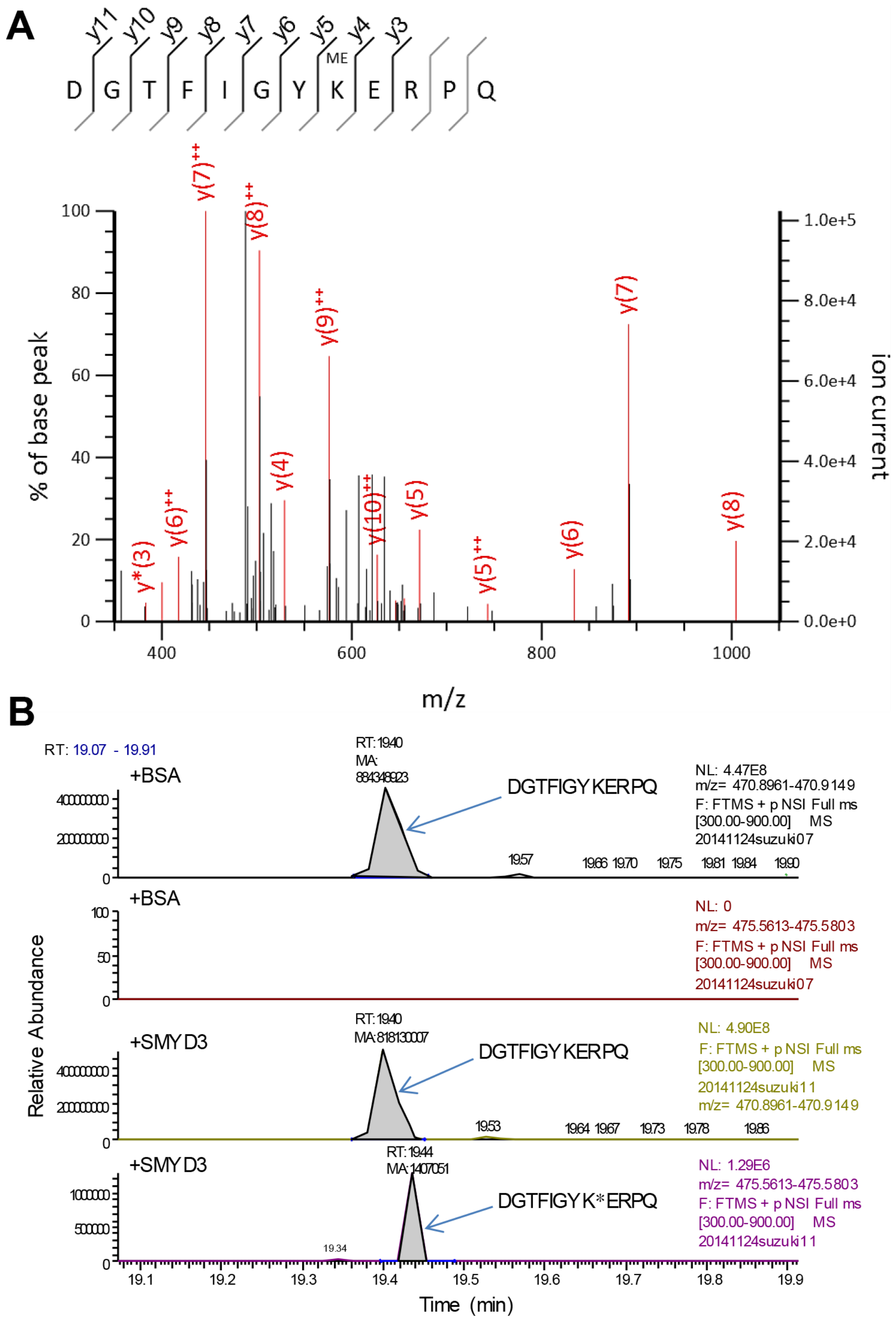

Supplementary Figure S5: Lysine 39 on AKT1 is methylated by SMYD3. A. The MS-MS spectrum corresponding to the monomethylated AKT1 32-43 peptide. The 14 Da increase of the lysine 39 residue was observed. Score and Expect show Mascot Ion Score and Expectation value in Mascot Database search results, respectively. **B.** MS chromatograms of unmodified and monomethylated AKT1 32-43 peptides.

A

#	b	b^{++}	b*	b^{*++}	b^0	b^{0++}	Seq.	y	y^{++}	y*	y^{*++}	y^0	y^{0++}	#
1	164.0706	82.5389					Y							14
2	**311.139**	156.0731					F	1529.836	765.4218	1512.81	756.9085	1511.826	756.4165	13
3	**424.2231**	212.6152					L	1382.768	691.8876	1365.741	683.3743	1364.757	682.8823	12
4	**537.3071**	269.1572					L	**1269.684**	635.3455	1252.657	626.8322	1251.673	626.3402	11
5	679.4178	340.2125	662.3912	331.6992			K	**1156.6**	578.8035	1139.573	570.2902	1138.589	569.7982	10
6	**793.4607**	397.234	776.4341	388.7207			N	**1014.489**	507.7482	997.4625	499.2349	996.4785	498.7429	9
7	**908.4876**	454.7475	891.4611	446.2342	890.4771	445.7422	D	900.4462	450.7267	883.4196	442.2134	882.4356	441.7214	8
8	**965.5091**	483.2582	948.4825	474.7449	947.4985	474.2529	G	**785.4192**	393.2132	768.3927	384.7	767.4087	384.208	7
9	**1066.557**	533.782	1049.53	525.2688	1048.546	524.7767	T	728.3978	364.7025	711.3712	356.1892	710.3872	355.6972	6
10	**1213.625**	607.3162	1196.599	598.803	1195.615	598.3109	F	**627.3501**	314.1787	610.3235	305.6654			5
11	**1326.709**	663.8583	1309.683	655.345	1308.699	654.853	I	**480.2817**	240.6445	463.2551	232.1312			4
12	1383.731	692.369	1366.704	683.8557	1365.72	683.3637	G	**367.1976**	184.1024	350.171	175.5892			3
13	1546.794	773.9007	1529.768	765.3874	1528.784	764.8954	Y	**310.1761**	155.5917	293.1496	147.0784			2
14							K	**147.1128**	74.06	130.0863	65.5468			1

B

#	b	b^{++}	b*	b^{*++}	b^0	b^{0++}	Seq.	y	y^{++}	y*	y^{*++}	y^0	y^{0++}	#
1	116.0342	58.5207			98.0237	49.5155	D							12
2	173.0557	87.0315			155.0451	78.0262	G	1309.69	655.3486	1292.663	646.8353	1291.679	646.3433	11
3	274.1034	137.5553			256.0928	128.55	T	1252.669	626.8379	1235.642	618.3246	1234.658	617.8326	10
4	421.1718	211.0895			403.1612	202.0842	F	1151.621	576.314	1134.594	567.8007	1133.61	567.3087	9
5	534.2558	267.6316			516.2453	258.6263	I	**1004.552**	502.7798	987.5258	494.2665	986.5418	493.7745	8
6	591.2773	296.1423			573.2667	287.137	G	**891.4683**	446.2378	874.4417	437.7245	873.4577	437.2325	7
7	754.3406	377.674			736.3301	368.6687	Y	**834.4468**	417.7271	817.4203	409.2138	816.4363	408.7218	6
8	896.4512	448.7293	879.4247	440.216	878.4407	439.724	K	**671.3835**	336.1954	654.357	327.6821	653.3729	327.1901	5
9	1025.494	513.2506	1008.467	504.7373	1007.483	504.2453	E	**529.2729**	265.1401	512.2463	256.6268	511.2623	256.1348	4
10	1181.595	591.3011	1164.568	582.7878	1163.584	582.2958	R	**400.2303**	200.6188	383.2037	192.1055			3
11	1278.648	639.8275	1261.621	631.3142	1260.637	630.8222	P	**244.1292**	122.5682	227.1026	114.055			2
12							Q	147.0764	74.0418	130.0499	65.5286			1

Supplementary Figure S6: MS/MS spectra of AKT1 peptides. LC-MS/MS analysis showed methylation of AKT1 at lysines 30 **A.** and 39 **B.** Theoretical values of MS fragments are summarized.

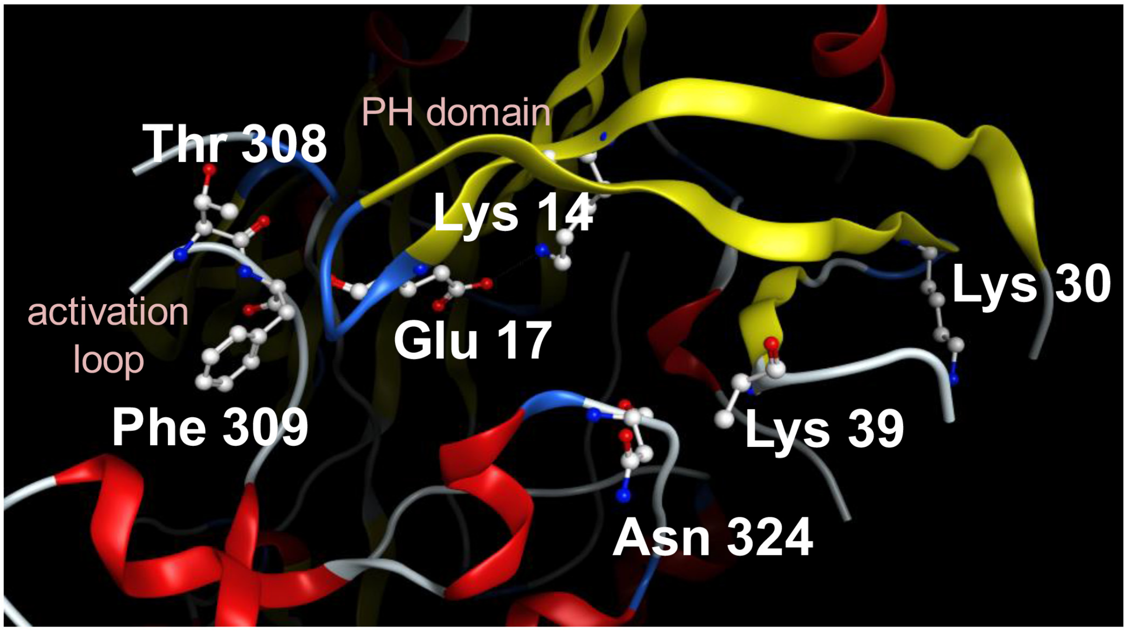

Supplementary Figure S7: Three dimensional structure of AKT1. The portions including three methylation sites (Lys 14, Lys 30 and Lys 39) in the PH domain and activation loop are described.

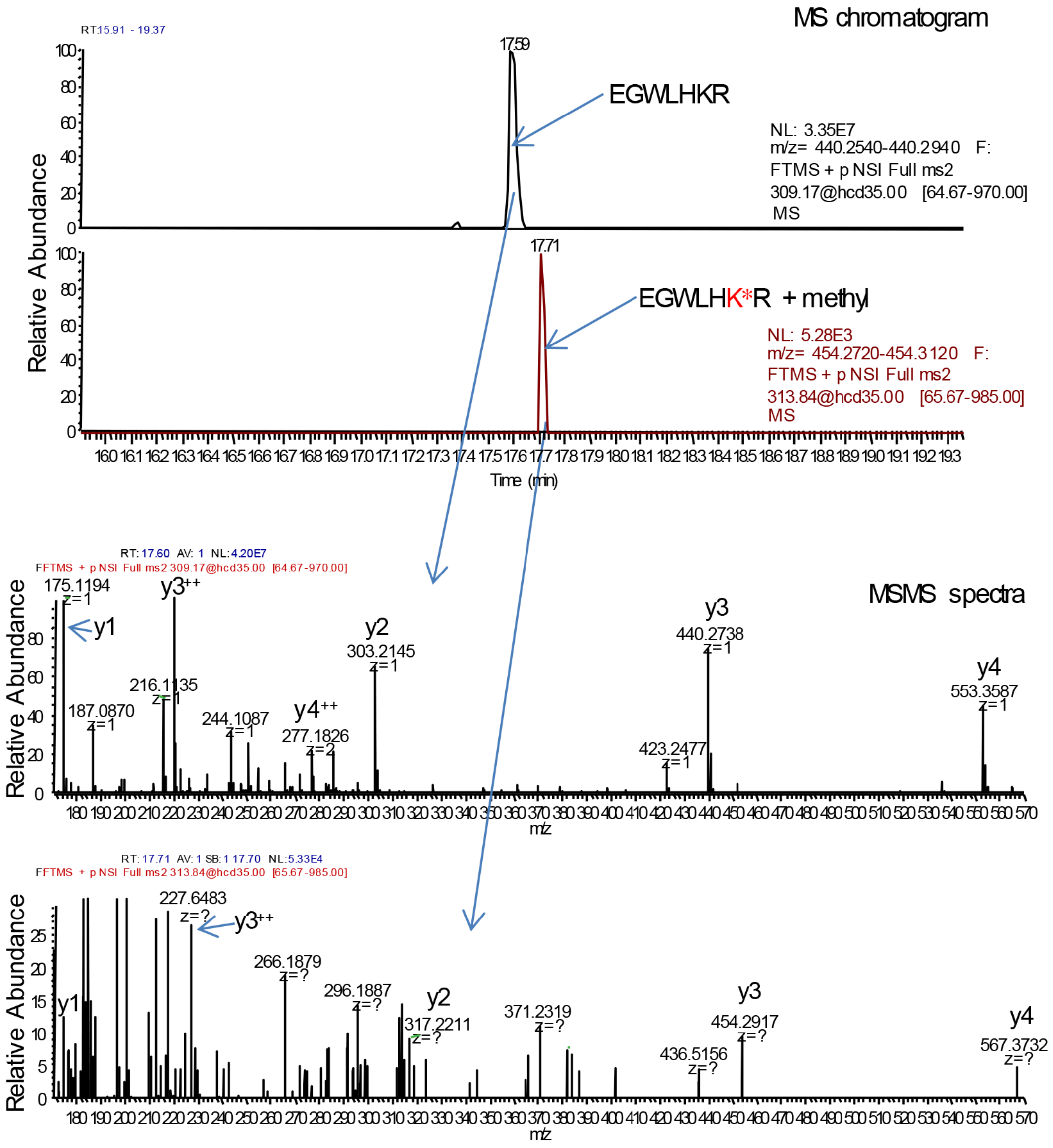

Supplementary Figure S8: Confirmation of *in vivo* methylation on AKT1 by LC-MS/MS analysis. Methylation of Lys 14 on AKT1 *in vivo* was confirmed by LC-MS/MS analysis. Wild-type FLAG-AKT1 and SMYD3 were co-overexpressed in 293T cells and purified by immunoprecipitation using anti-FLAG® M2 affinity gel, followed by SDS-PAGE. Separated AKT1 protein was excised and digested in-gel with trypsin, and generated peptides were analyzed by LC-MS/MS. Monomethylation of Lys 14 on AKT1 was detected.

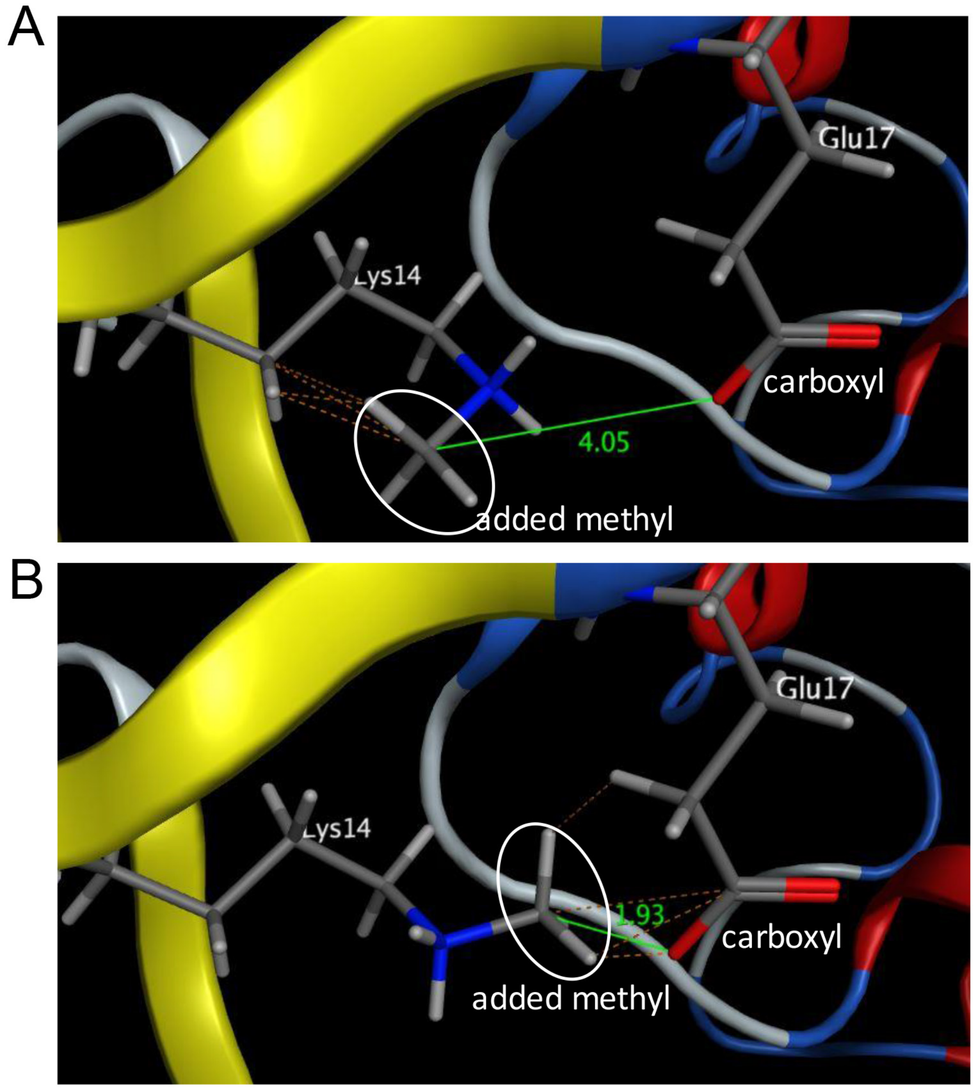

Supplementary Figure S9: Three dimensional structure around Lys 14 of AKT1. The methyl located at the maximum distance (4.05 Å) **A.** and the minimum distance (1.93 Å) **B.** is shown.

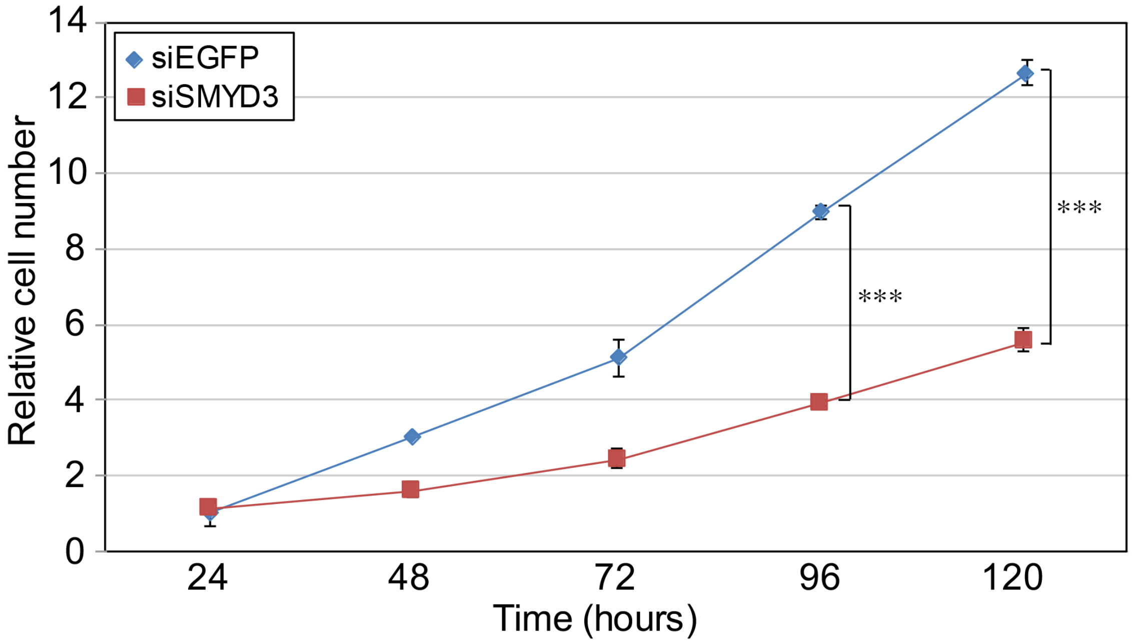

Supplementary Figure S10: Cell growth assays of SW480 cells after treatment with siEGFP and siSMYD3. SW480 cells were treated with siEGFP or siSMYD3#2, and the cell number was measured with CCK-8 (Dojindo) 24, 48, 72, 96 or 120 hours after treatment with siRNAs. The relative cell number shows relative value of cell number compared to the cell number at 24 h. Results are the means ± s.d. of three independent cells. *P*-values were calculated using Student's *t*-test (***, $P < 0.001$).

Supplementary Table S1: Information of certificated cell lines

Name	Origin	Certification institution	Tested method	DNA profile or characteristics
SW480	human colorectal adenocarcinoma	ATCC	STR	Amelogenin: X CSF1PO: 13,14 D13S317: 12 D16S539: 13 D5S818: 13 D7S820: 8 THO1: 8 TPOX: 11 vWA: 16
MDA-MB-231	human breast cancer	ATCC	STR	Amelogenin: X, X CSF1PO:12,13 D13S317:13 D16S539:12 D5S818:12 D7S820:8,9 TH01:7,9.3 TPOX:8,9 vWA:15,19 D3S1358: 16 D21S11: 30,33.2 D18S51: 11,16 Penta E: 11 Penta D: 11,14 D8S1179: 13 FGA: 22,23
293T	human embryonic kidney fibroblast	ATCC	STR	Amelogenin: X CSF1PO: 11, 12 D13S317: 12, 14 D16S539: 9, 13 D5S818: 8, 9 D7S820: 11 THO1: 7, 9.3 TPOX: 11 vWA: 16, 18, 19
HeLa	human cervix carcinoma	ATCC	STR	Amelogenin: X, Y CSF1PO: 11,12 D13S317: 11,14 D16S539: 9,11 D5S818: 11,12 D7S820: 10,11 THO1: 8 TPOX: 8 vWA: 15

ATCC; American Type Culture Collection.

Supplementary Table S2: siRNA sequences

siRNA name	Sequence
siEGFP	Sense: 5' GCAGCACGACUUCUUCAAG 3'
	Antisense: 5' CUUGAAGAAGUCGUGCUGC 3'
siSMYD3#1	Sense: 5' GAUUGAAGAUUUGAUUCUA 3'
	Antisense: 5' UAGAAUCAAAUCUUCAAUC 3'
siSMYD3#2	Sense: 5' CAGCAAUUCUGAACGGCUU 3'
	Antisense: 5' AAGCCGUUCAGAAUUGCUG 3'
